# Rapid exploration of anti-MRSA components from plants of *Salvia*

**DOI:** 10.1007/s13659-026-00624-0

**Published:** 2026-06-01

**Authors:** Huan Huang, Zhao-Jie Wang, Li-Yu Bai, Yue-Ming Jiang, Yan-Yan Zhu, Yun-Li Zhao, Xiao-Dong Luo

**Affiliations:** 1 Yunnan Characteristic Plant Extraction Laboratory, Key Laboratory of Medicinal Chemistry for Natural Resource, Ministry of Education and Yunnan Province, School of Chemical Science and Technology, Yunnan University, Kunming, 650500 People’s Republic of China; 2 State Key Laboratory of Phytochemistry and Plant Resources in West China, Kunming Institute of Botany, Chinese Academy of Sciences, Kunming, 650201 People’s Republic of China

**Keywords:** *Salvia*, Anti-MRSA, Miltirone, Przewaquinone A, Mechanism, In vitro and in vivo

## Abstract

Plants of *Salvia* are frequently used for treatment infections of boils and sores in Traditional Chinese medicine, which might inhibit microbial. In this work, the chemical constituents of the effective fractions were analyzed using UPLC-Q-TOF–MS, and multivariate analysis and network pharmacology were used to predict potential antibacterial substances and their mechanism. Moreover, against methicillin-resistant *Staphylococcus aureus* (MRSA) effect and pathway of bioactive compounds were validated by experiments in vitro and in vivo. Two unreported anti-MRSA compounds, miltirone and przewaquinone A were proposed and then validated by serial experiments. They acted as bactericides and biofilm scavengers, respectively, and both targeted on the cell membrane, causing leakage of contents and affecting intracellular metabolism, leading to bacterial death without significant toxicity. Furthermore, miltirone ameliorated skin wound infection caused by MRSA in mice, roughly equal to vancomycin. The research supported the traditional use of *Salvia* plants, and presented two potent bactericidal agents for further investigation.

**Graphical Abstract:**

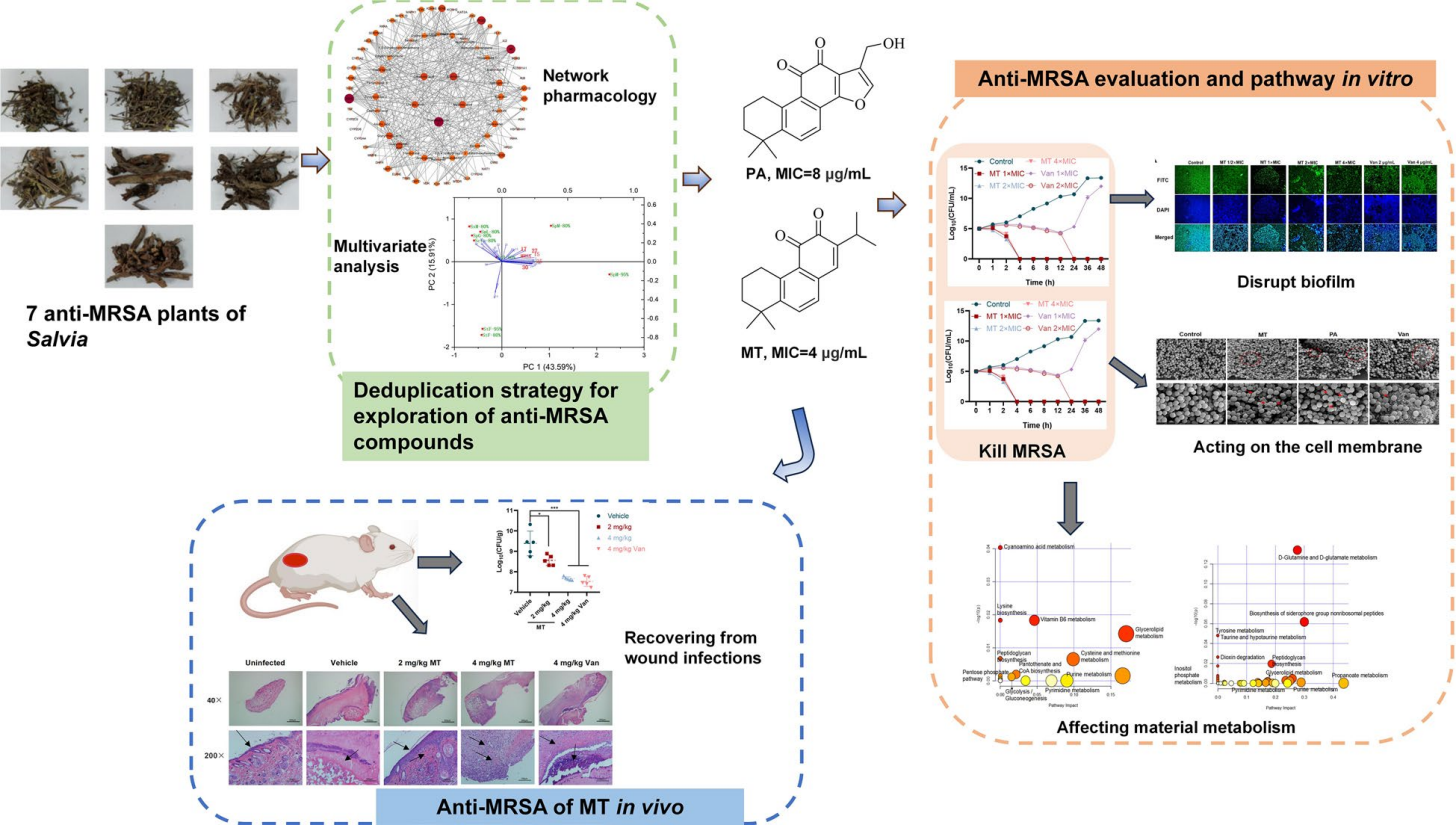

**Supplementary Information:**

The online version contains supplementary material available at 10.1007/s13659-026-00624-0.

## Introduction

*Staphylococcus aureus* is a common gram-positive pathogen [1, 2] that imposes a substantial healthcare burden, with tens of thousands of annual deaths attributed to *S. aureus* infections [3]. High mortality and incidence rates of bacteremia, bone infection, and endocarditis can be attributed to this factor. MRSA has high levels of resistance, and the efficacy of most antibiotics currently available in clinical practice is progressively decreasing [4]. In 2019, the direct impact of MRSA resulted in an estimated 10,000 fatalities [5]. Plants natural products are a crucial source of anti-MRSA drugs [6] and are an important strategy for overcoming bacterial resistance [7]. Then there is a high level of expectation for the extensive application of plant-derived antibacterial agents as effective remedies to address the current challenges of antibiotic resistance.

The *Salvia* genus of the Labiatae family is distributed mainly in southwest China [8]. The chemical constituents of this genus, including diterpenoids, triterpenoids, flavonoids and steroids exhibited therapeutic properties, such as anti-inflammatory, detumescent and efficacy against dysentery in Materia Medica (Ben-Cao-Shi-Yi in Chinese), which might be caused by *S. aureus* [9]. The anti-MRSA potential of seven plants was screened (Table S1), and different extracts of 7 *Salvia* plants showed anti-MRSA bioactivity with minimum inhibitory concentrations (MIC) ranging from 16 to 256 μg/ml (Table S2), which supposed the presence of same bioactive compounds in this genus. To avoid repeating isolation of the seven plants routinely, chemical compositions analysis of the bioactive fractions from each plants were carried out by UPLC-Q-TOF–MS, and then anti-MRSA bioactive compounds were predicted using multivariate analysis [10] and network pharmacology [11]. As a result, two unknown anti-MRSA compounds, miltirone (MT) and przewaquinone A (PA) were explored, and their anti-MRSA efficiency and pathway were validated in vitro and in vivo.

## Results

### Identification and quantification of compounds from bioactive plants

**Fig. 1:**
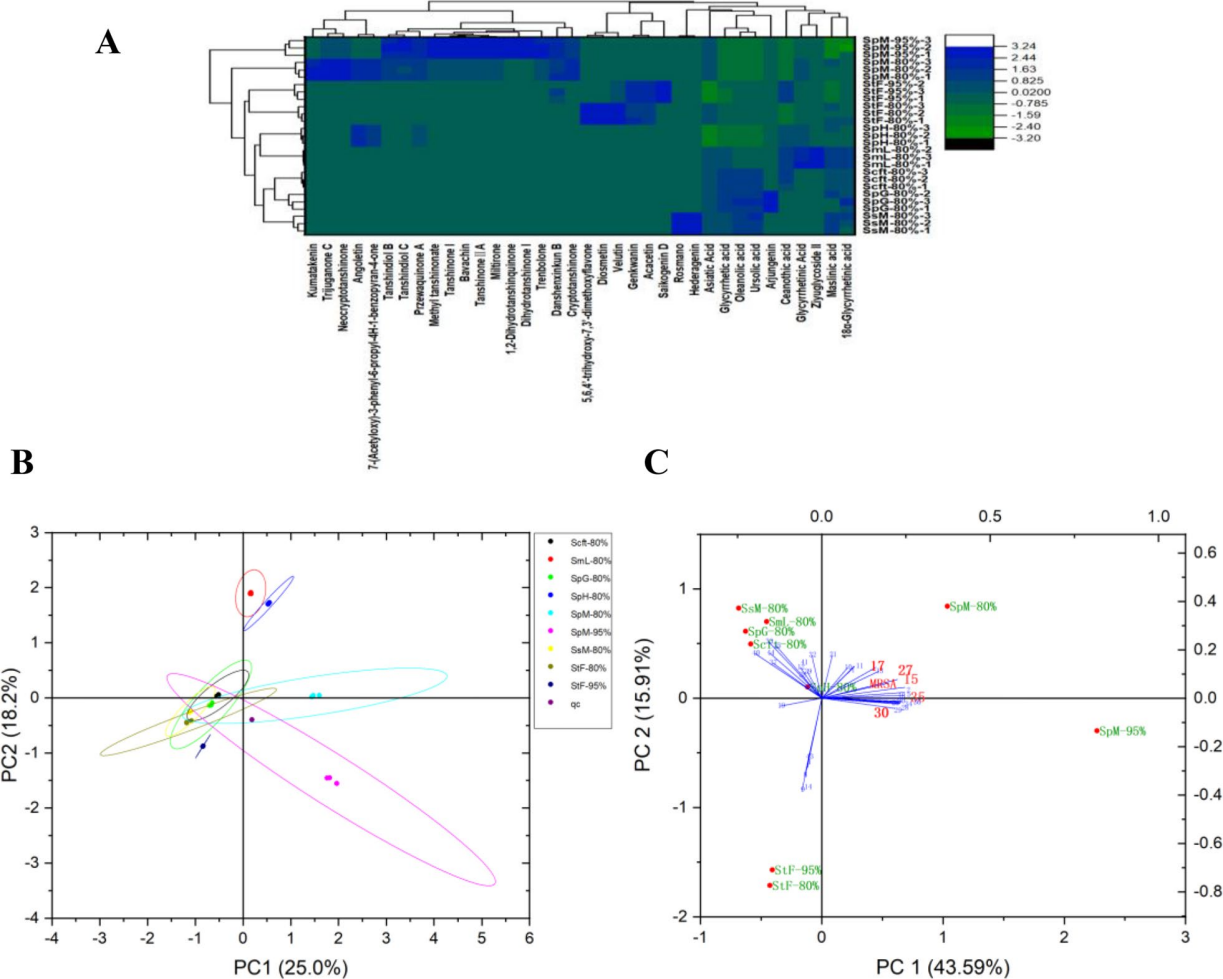
**A** Heatmap. **B** Score plots. **C** Principal component analysis of correlations between compounds and anti-MRSA bioactivity

9 bioactive fractions from 7 *Salvia* plants were analyzed using UPLC-Q-TOF–MS, and the results of the TICs were shown in Fig. S1A and B. QC samples were used to assess the stability of the instrument, and three biological replicates in each group showed good repeatability (Fig. 1B). A total of 44 compounds were identified (Table 1), including 15 diterpenes, 9 flavonoids, and 13 triterpenes, in which 36 major compounds were quantified (Table S5). MS^1^ exists mainly in the form of [M + H]^+^, and the fragmentation pathway of przewaquinone A (**15**) was examined as an example (Fig. S3). The limits of detection (LOD) and quantification (LOQ) in Table S4 were determined using signal-to-noise ratios (S/N) of 3 and 10, respectively. The differences among the 9 fractions were visualized using the heatmap (Fig. 1A), and two principal components (Fig. 1B, C) explained 59.5% of the total variability. Then przewaquinone A (**15**), neocryptotanshinone (**17**), cryptotanshinone (**27**), dihydrotanshinone I (**30**), and miltirone (**35**) showed highly correlated to anti-MRSA bioactivity by PCA analysis.

**Table 1:** Chemical constituents by UPLC-Q-TOF–MS analysis

No	RT (time)	ESI mode	Err (ppm)	m/z	MS/MS fragment ions	Molecular Formula	Compounds	References
1	4.45	[M + H]^+^	− 0.95	315.0873	299.05, 257.04, 121.02	C_17_H_14_O_6_	Kumatakenin	Standard
2	4.60	[M + H]^+^	3.19	313.1081	373.16, 295.09, 267.10	C_18_H_16_O_5_	Tanshindiol B	[12]
3	4.83	[M-H]^−^	2.23	359.0780	161.02, 197.04, 135.04, 72.99	C_18_H_16_O_8_	Rosmarinic acid	MSDIAL
4	4.89	[M + H]^+^	2.11	331.0819	331.08	C_17_H_14_O_7_	5,6,4’-trihydroxy-7,3’- dimethoxyflavone	Standard
5	4.90	[M + H]^+^	2.87	313.1080	295.09, 267.10	C_18_H_16_O_5_	Tanshindiol C	MSDIAL, GNPS
6	5.04	[M + Na]^+^	1.22	329.1388	229.08, 197.05, 169.06, 141.06,	C_19_H_20_O_5_	Columbianadin	MSDIAL
7	5.19	[M + H]^+^	0.33	301.0708	258.05, 186.98, 163.03, 124.01	C_16_H_12_O_6_	Diosmetin	MSDIAL
8	5.33	[M + H]^+^	0.95	315.0866	300.06, 282.05, 187.14, 108.02	C_17_H_14_O_6_	Velutin	MSDIAL
9	5.81	[M + H]^+^	7.37	285.0778	242.05, 229.08, 187.03, 124.01	C_16_H_12_O_5_	Genkwanin	MSDIAL
10	6.11	[M + H]^+^	1.33	301.1438	283.13, 268.10, 265.12, 185.05	C_18_H_20_O_4_	Angoletin	MSDIAL GNPS
11	6.11	[M + Na]^+^	− 7.43	323.1254	309.10, 265.08, 250.10, 223.07	C_20_H_18_O_4_	7-(Acetyloxy)-3-phenyl-6-propyl-4H-1-benzopyran-4-one	MSDIAL
12	6.48	[M-H]^−^	3.77	345.1720	283.17, 227.10, 268.14	C_20_H_26_O_5_	Rosmanol	Standard
13	6.66	[M + H]^+^	4.10	341.1398	313.14, 281.08, 263.07	C_20_H_20_O_5_	Trijuganone C	[13]
14	7.23	[M-H]^−^	3.18	283.0621	268.03, 240.09, 151.00, 117.03, 107.01	C_16_H_12_O_5_	Acacetin	MSDIAL [14]
15	7.23	[M + H]^+^	− 2.57	311.1278	293.11, 275.10, 265.12	C_19_H_18_O_4_	Przewaquinone A	Standard
16	7.33	[M + H]^+^	1.80	389.1966	329.17, 311.16, 287.16	C_22_H_28_O_6_	Quassin	MSDIAL
17	7.57	[M + H]^+^	1.90	315.1597	297.15, 279.13, 254.09, 193.10	C_19_H_22_O_4_	Neocryptotanshinone	Standard
18	7.84	[M + H]^+^	7.08	339.1251	311.12, 279.10, 261.09, 233.09	C_20_H_18_O_5_	Methyl tanshinonate	[15]
19	7.86	[M + H]^+^	0.21	487.3419	469.33, 423.32, 405.31, 165.06, 119.08	C_30_H_46_O_5_	Ceanothic acid	MSDIAL
20	7.95	[M + H]^+^	0.00	281.1172	303.09, 235.11	C_18_H_16_O_3_	Danshenxinkun B	MSDIAL [16]
21	8.13	[M-H]^−^	0.82	487.3433	487.34, 227.21	C_30_H_48_O_5_	Asiatic Acid	MADIAL
22	8.22	[M + H]^+^	0.00	279.2319	262.22, 233.09, 95.08, 81.07, 67.05	C_18_H_30_O_2_	Linolenic acid	MSDIAL
23	8.50	[M + H]^+^	3.32	301.2172	259.16, 213.12, 163.07, 171.08	C_20_H_28_O_2_	Sugiol	[13]
24	8.68	[M-H]^−^	2.43	329.1766	285.18, 201.09	C_20_H_26_O_4_	Carnosol	Standard
25	8.69	[M + H]^+^	− 1.91	471.3469	453.23, 425.34, 189.16, 175.14, 95.08	C_30_H_46_O_4_	Glycyrrhetic acid	MSDIAL [17]
26	8.78	[M + H]^+^	3.05	277.0873	249.09, 178.07,141.06	C_18_H_12_O_3_	Tanshinone I	Standard
27	8.98	[M + H]^+^	2.39	297.1504	254.09, 221.09, 193.10	C_19_H_20_O_3_	Cryptotanshinone	Standard [18]
28	9.18	[M + Na]^+^	2.46	325.1442	297.14, 269.15, 193.100, 167.08,	C_20_H_20_O_4_	Bavachin	MSDIAL
29	9.51	[M + H]^+^	4.30	279.1028	261.09, 233.09, 205.10, 190.07	C_18_H_14_O_3_	1,2-Dihydrotanshinquinone	Standard
30	9.52	[M + H]^+^	4.66	279.1029	233.09, 261.09, 205.10	C_18_H_14_O_3_	Dihydrotanshinone I	Standard
31	9.68	[M-H]^−^	2.78	503.3392	-	C_30_H_48_O_6_	Pygenic acid C	MSDIAL
32	10.12	[M + H]^+^	4.24	471.3489	407.33, 247.16, 201.16, 119.08	C_30_H_46_O_4_	Glycyrrhetinic Acid	MSDIAL
33	10.41	[M-H]^−^	0.42	471.3482	471.34, 405.30	C_30_H_48_O_4_	Maslinic acid	Standard
34	10.52	[M + H]^+^	5.42	295.1345	277.12, 253.11, 46.06	C_19_H_18_O_3_	Tanshinone IIA	Standard
35	10.95	[M + H]^+^	1.77	283.1698	223.11, 179.08, 165.07	C_19_H_22_O_2_	Miltirone	Standard
36	11.79	[M + H]^+^	2.21	271.1699	241.12, 227.10, 199.07, 117.07	C_18_H_22_O_2_	Trenbolone	MSDIAL [19]
37	12.79	[M + H]^+^	0.44	457.3676	–	C_30_H_48_O_3_	Oleanolic acid	MSDIAL, GNPS
38	12.79	[M + Na]^+^	4.55	505.3547	–	C_30_H_48_O_6_	Arjungenin	MSDIAL
39	12.87	[M + Na]^+^	2.87	627.3685	–	C_35_H_56_O_8_	Ziyuglycoside II	MSDIAL
40	12.92	[M-H]^−^	1.70	469.3331	–	C_30_H_46_O_4_	18α-Glycyrrhetinic acid	MSDIAL
41	13.37	[M-H]^−^	2.76	471.3491	–	C_30_H_48_O_4_	Hederagenin	MSDIAL
42	13.42	[M-H]^−^	2.33	471.3491	471.34, 423.32	C_30_H_48_O_4_	Pygenic acid A	MSDIAL
43	14.17	[M + Na]^+^	− 3.23	495.3445	435.23, 205.15, 101.07, 45.03	C_30_H_48_O_4_	Saikogenin D	MSDIAL
44	14.60	[M-H]^−^	1.98	455.3540	407.33, 297.23, 48.82	C_30_H_48_O_3_	Ursolic acid	Standard

*RT* retention time

### Prediction of bioactivity components and mechanism by network pharmacology

**Fig. 2:**
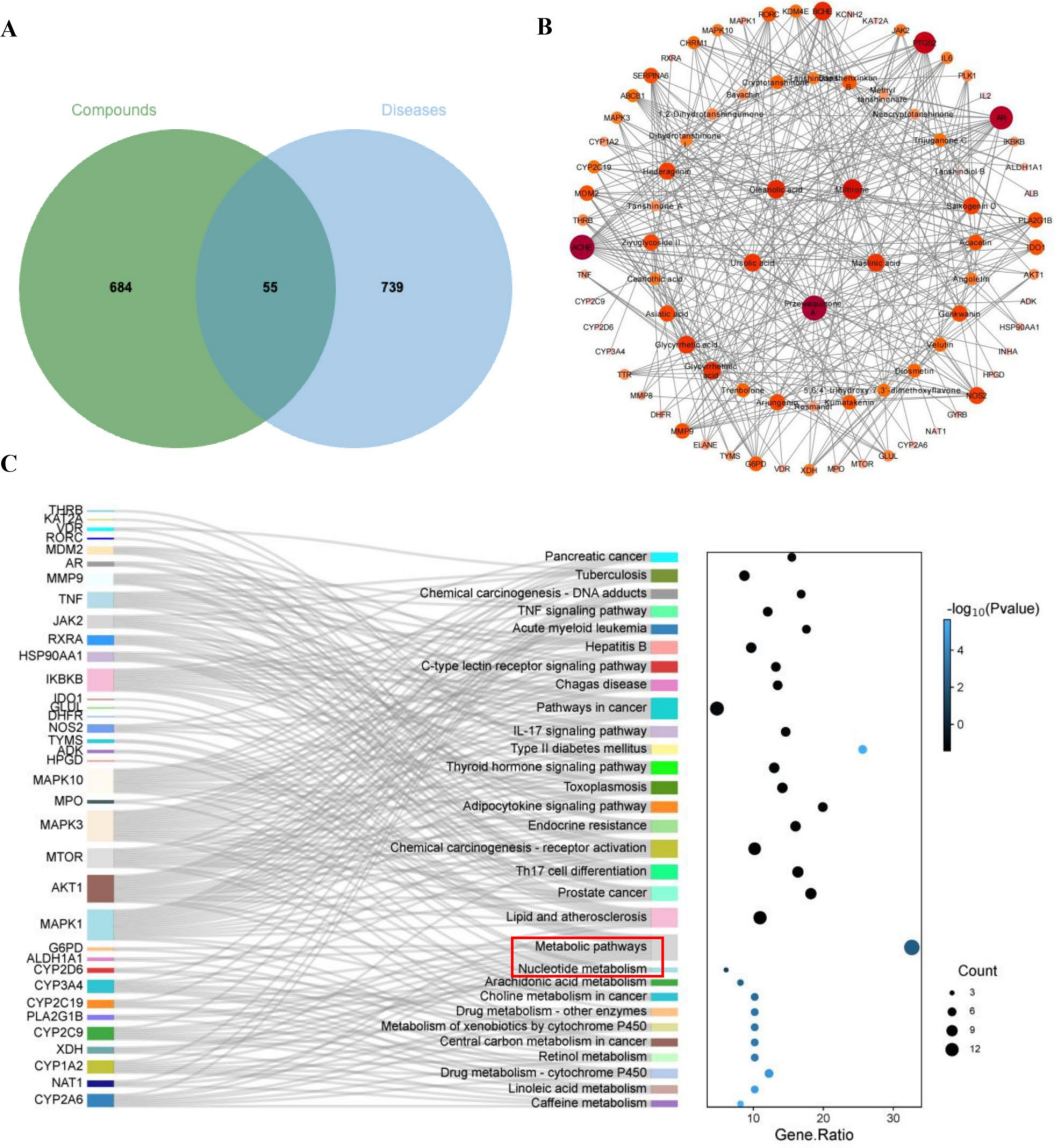
**A** The Venn graph of the main components targets of biological fractions and anti-MRSA targets. **B** Potential effective compounds against MRSA. The color depth represented the importance of targets. **C** Sankey bubble chart of KEGG enrichment analysis

The database predicted 739 targets for the 36 compounds and 794 targets for the anti-MRSA antibodies. Venn analysis revealed an overlap of 55 targets, indicating potential therapeutic targets (Fig. 2A). The protein–protein interaction (PPI) network (Fig. S4A) was constructed by importing these targets into the STRING database and visualizing them using Cytoscape 3.7.1., and then “disease-targeted drug target” network was constructed (Fig. 2B) to present common targets for compounds and diseases and predict potential bioactive compounds [11]. As a result, przewaquinone A (**15**), miltirone (**35**), oleanolic acid (**37**), maslinic acid (**33**), and ursolic acid (**44**) were supposed to be bioactive components, in which unreported przewaquinone A (**15**) and miltirone (**35**) were selected for further investigation. Moreover, the KEGG analysis (Fig. 2C) showed that the main targets were enriched in nucleotide metabolism, linoleic acid metabolism, and arachidonic acid metabolism.

### Miltirone and przewaquinone A acted as bactericide and removing biofilm

**Fig. 3:**
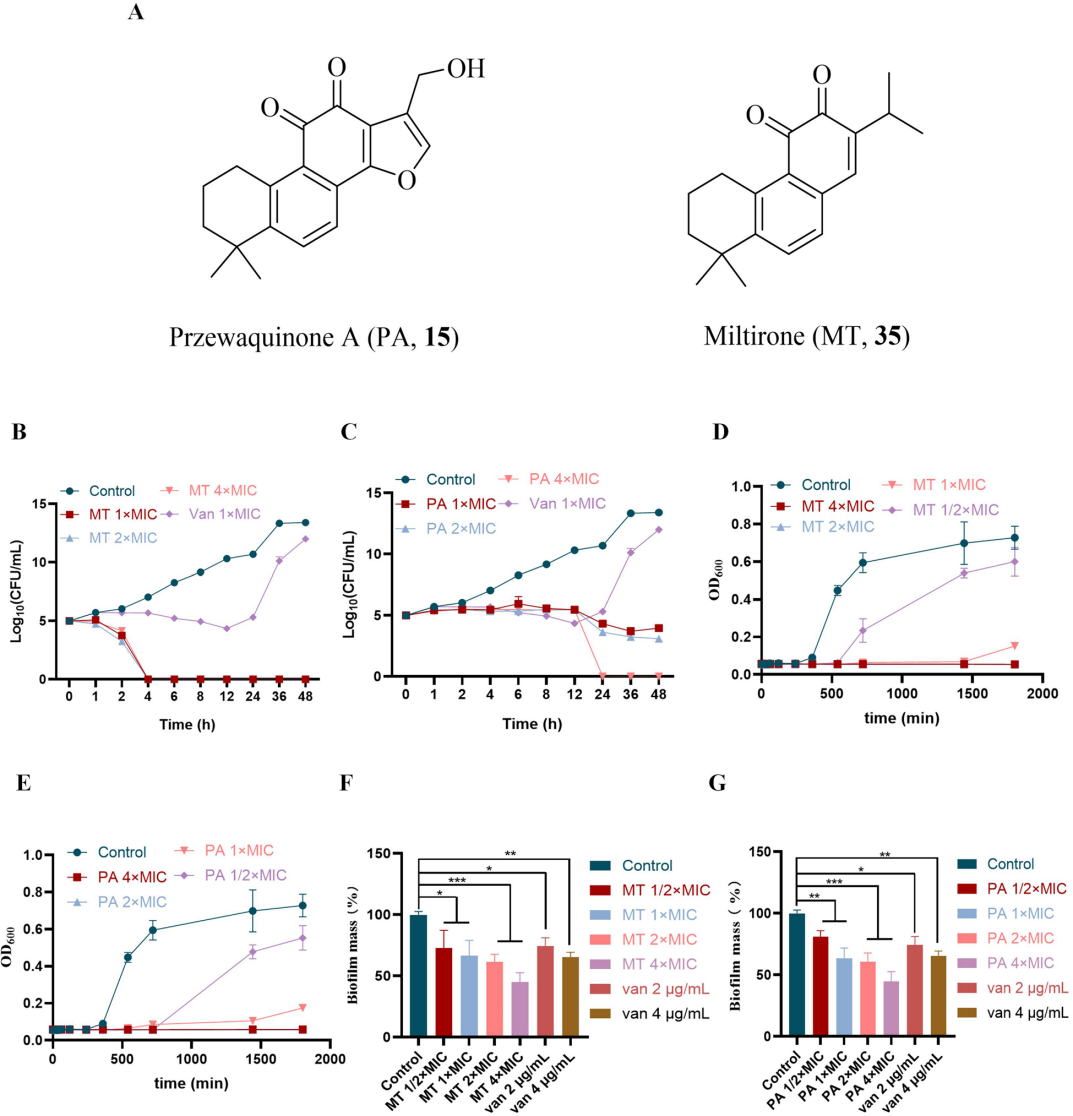
**A** Structures of przewaquinone A (**15**) and miltirone (**35**). **B**, **C** Time–kill curve 1 – 4 × MIC. **D**, **E** Growth of exponential phase cultures 1/2–4 × MIC. **F**, **G** Effect of 1/2–4 × MIC on preformed biofilm. The data in the figure is represented by “mean ± standard deviation”, with three replicates in each group. ****P < 0.0001 vs control; ***P < 0.001 vs control; **P < 0.01 vs control; *P < 0.05 vs control

Przewaquinone A and miltirone showed good antibacterial bioactivity against multidrug-resistant gram-positive bacteria (Table S6) particularly against MRSA, and then a time-kill curve was used to evaluate the sterilization performances of them at 48 h (Fig. 3B, C). Miltirone exhibited excellent bactericidal bioactivity with the bacterial count under the detection limit at 1 × , 2 × , and 4 × MIC after 4 h, while przewaquinone A completely killed the bacteria at an MIC of 4 × after 24 h, showing advantage over vancomycin. In addition, both compounds inhibited growth of MRSA in a dose-dependent manner (Fig. 3D, E). Moreover, they showed scavenging effect on biofilm formation with best clearance effect of 55% at 4 × MIC (16 μg/mL) for miltirone and 32 μg/mL for przewaquinone A (Fig. 3F, G).

**Fig. 4:**
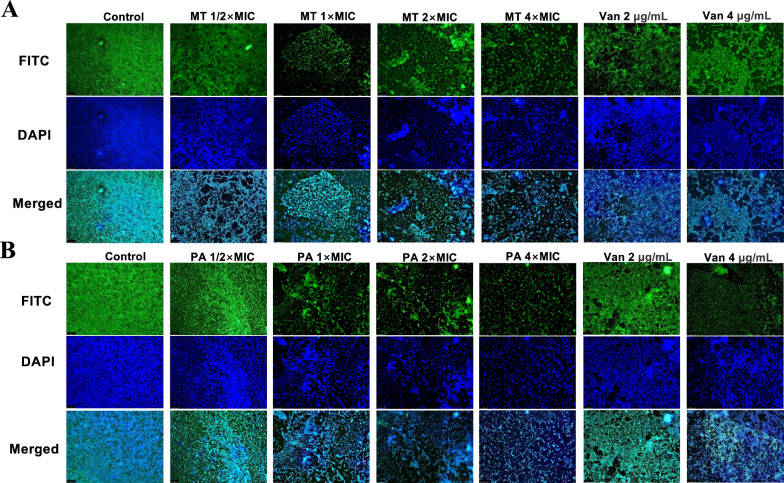
**A**, **B** FM images of the MRSA00 biofilm

Furthermore, the fluorescence microscope (FM) images showed similar results with the biofilm destroyed dependent on doses (Fig. 4A, B), in which FITC binded to proteins and nucleic acids on the bacterial surface and DAPI binded to DNA.

### Miltirone and przewaquinone A targeted on cell membrane and causing leakage of contents without toxicity

**Fig. 5:**
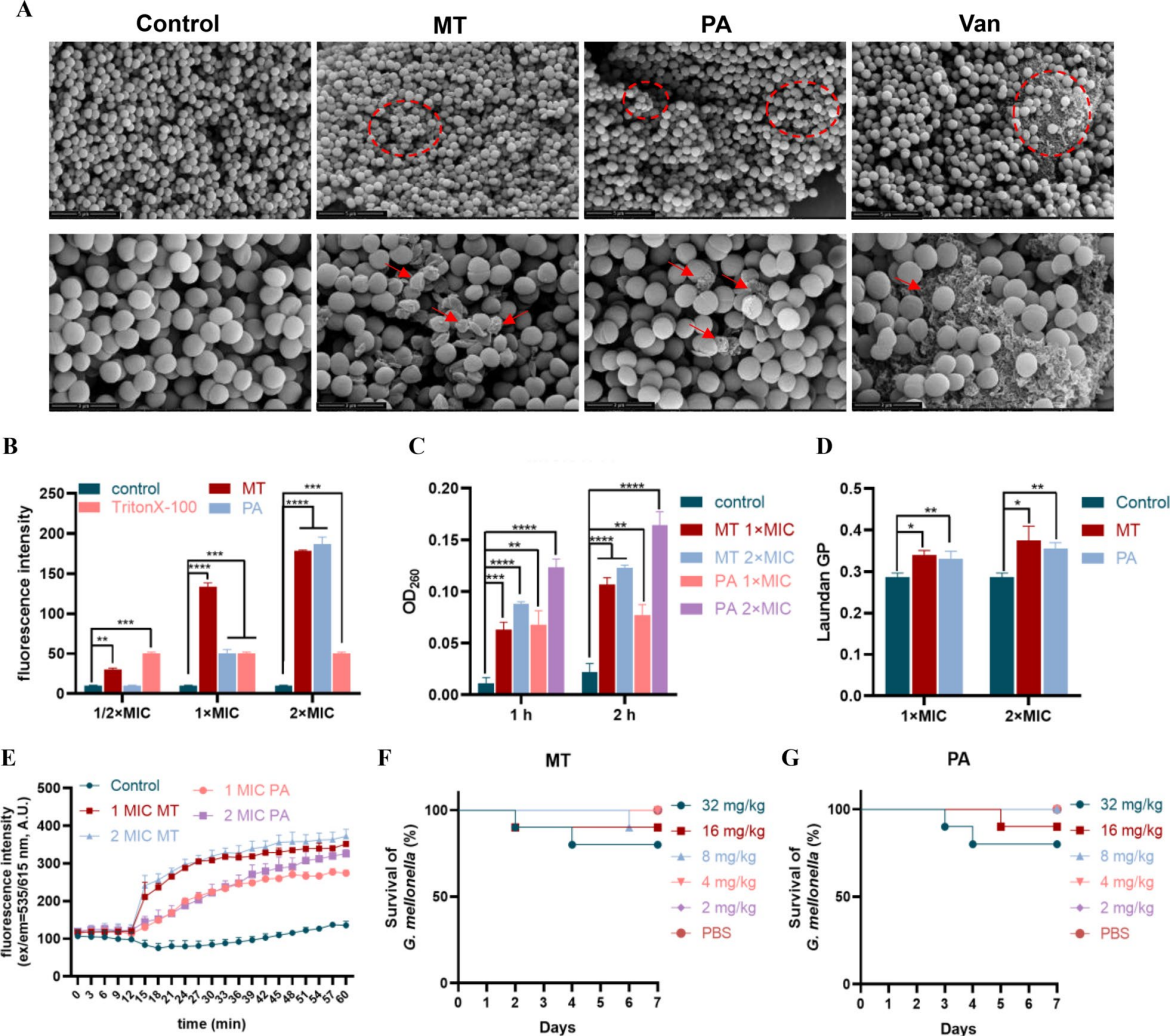
**A** Effect of 4 × MIC miltirone or przewaquinone A and vancomycin on MRSA003 biofilm bacterial morphology. Scale bars in the overall and magnified images represent 5 μm and 2 μm, respectively. **B** Compounds (1/2–2 × MIC) increasing the membrane permeability of MRSA003. **C** Amount of nucleic acid leakage. D: Reducing the membrane fluidity of MRSA003. E: The membrane potential of MRSA003 after treatment. **F**–**G** The survival rate of *G. mellonella* larvae after compounds treatment. The data in the figure represented by “mean ± standard deviation”, with three replicates in each group. ****P < 0.0001 vs control; ***P < 0.001 vs control; **P < 0.01 vs control; *P < 0.05 vs control

MRSA003 exhibited a smooth surface and normal morphology in the control group, but showed shriveled and ruptured surfaces by miltirone and przewaquinone A treatment (Fig. 5). Then the impact of them on the integrity of cell membranes were further evaluated by measuring fluorescence intensity (Fig. 5B), which indicated the effects of miltirone and przewaquinone A on cell membrane integrity in a dose-dependent manner. Furthermore, they induced the release of *β*-galactosidase from MRSA003, dependent on time and concentration (Fig. 5C). In addition, membrane fluidity were changed by both compounds from fluorescent probe Laurdan (Fig. 5D), indicating disruption of basic metabolic and bacterial homeostasis. The fluorescence intensity were increased after their treatment in a dose-dependent manner (Fig. 5E), suggesting both compounds disruption of the internal positive and external negative polarization states of cell membranes, and then leading to changes in the permeability of the cell membrane. Finally, miltirone and przewaquinone A did not show any toxicity to *Galleria mellonella* larvae at treatment concentrations (Fig. 5F–G).

### Effects of miltirone and przewaquinone A on the metabolome of MRSA

**Fig. 6:**
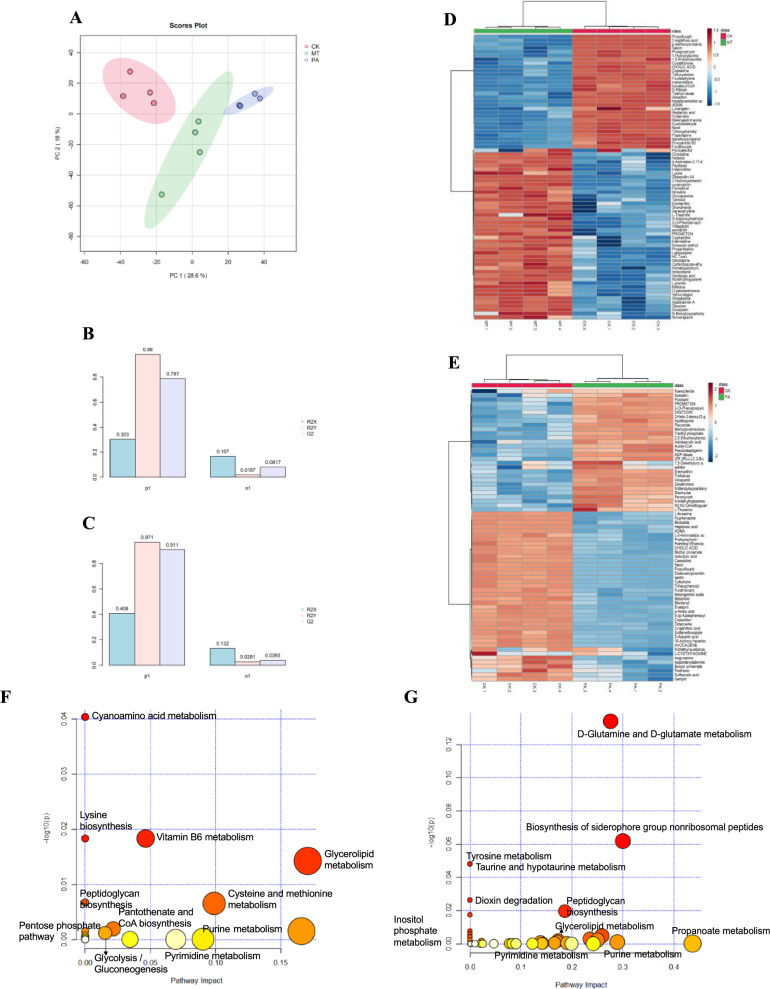
Effects of miltirone and przewaquinone A on the metabolome of MRSA003. **A** Principal component analysis of the metabolome resulted in a significant separation of three groups. **B**, **C** The model validation plots (miltirone and przewaquinone A). **D**–**E** Heat-map and hierarchical clustering analysis of the metabolite differentially abundant in MRSA003 treated with miltirone (**D**) or przewaquinone A (**E**) (FDR < 0.05)). **F**, **G** KEGG enrichment analysis of metabolic pathways based differential metabolites in MRSA treated with miltirone (**F**) or przewaquinone A (**G**)

To elucidate underlying mechanism of miltirone and przewaquinone A, intracellular metabolomic was performed using UPLC-Q-TOF–MS, and PCA was used to conduct overall metabolic variation between samples and within each group. The PCA results showed differences in the metabolic groups (Fig. 6A), and the validation miltirone (Fig. 6B) and przewaquinone A (Fig. 6C) model were shown as follow: R2X = 0.47 and 0.54, R2Y = 0.9987 and 0.9991, Q2 = 0.8687 and 0.9503, respectively. 159 metabolites were altered significantly with 139 up-regulated and 20 down-regulated in the miltirone group, while 96 metabolites were altered significantly with 60 metabolites up-regulated and 20 metabolites down-regulated (VIP > 1.5, FDR < 0.05)) in the przewaquinone A group. Hierarchical clustering was performed based on the top 100 differential metabolites with VIP values to observe changes in metabolites and draw a clustering heatmap for the miltirone (Fig. 6D) and przewaquinone A groups (Fig. 6E). KEGG enrichment analysis (Fig. 6F, G) suggested their influence on a wide range of metabolic pathways in MRSA.

Phosphatidylglycerol is the main component of the membrane of MRSA [20]. As shown in Fig. 5F, G, MT and PA can affect glycerolipid metabolism, which can destroy membranes and inhibit repair. This is consistent with the previous experimental results. Besides, MT and PA can affect the metabolism of multiple amino acids including cyanoamino acid metabolism, lysine biosynthesis, tyrosine metabolism, d-glutamine and d-glutamate metabolism. Amino acids are the fundamental units that make up proteins and are crucial for bacterial growth [21]. The metabolism of amino acids is affected which will affect the synthesis and repair of proteins, as well as the production of carbon and nitrogen sources within the bacteria, thereby affecting bacterial growth.

Carbohydrate metabolism is an important pathway for the energy required for bacterial activity [22]. MT and PA can impact glycolysis gluconeogenesis and various other energy metabolism pathways in this article. Specifically, changes in the external environment can also lead to disturbances in energy metabolism [22]. No matter what the reason is, some changes caused by energy metabolism disorders were found in this article. In addition, some pathways related to nucleotide metabolism are also affected such as pyrimidine metabolism and purine metabolism, which is consistent with the results of network pharmacology.

### *Antibacterial effect *in vivo

**Fig. 7:**
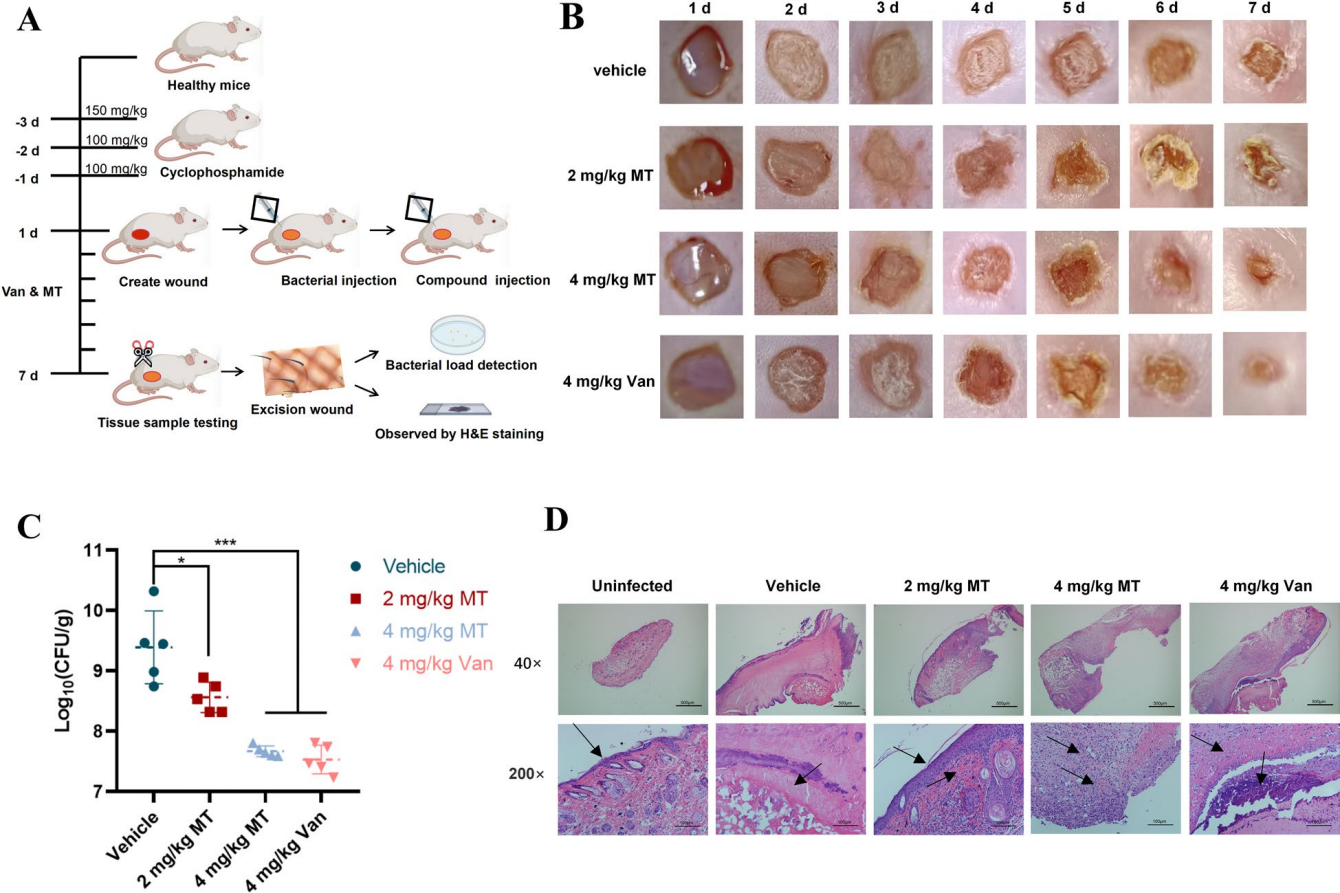
**A** Schematic diagram of mice skin wound infection model. **B** Representative photographs of the wound changes of mice under the treatment of various concentrations of miltirone (2 mg/kg and 4 mg/kg) and vancomycin. **C** The bacterial load in mice skin wounds. The data in the figure represented by “mean ± standard deviation”, with five replicates in each group. **D** Representative wound tissue histopathology based on H&E staining

Wound infection model was established to evaluate antibacterial effect miltirone in vivo (Fig. 7A), and as a result, the wound sizes were significantly reduced in the groups of 4 mg/kg and 2 mg/kg, respectively, in seven days miltirone treatment (Fig. 7B). Meanwhile, the bacterial load were reduced by miltirone treatment, roughly equal to vancomycin at same dose (Fig. 7C). In addition, the epidermis of healthy skin tissue was intact with a clear structure, and the collagen fibers in the dermis were neatly organized (Fig. 7D), but widespread necrosis of skin tissue was observed as an unstructured eosinophilic substance in the model group. Significant improvement in the skin by miltirone or vancomycin treatment were observed, with neovascularization in the dermis and subcutaneous tissue, and the thickness of surrounding epidermis at the injury sites.

## Discussion

*Salvia* species are an important resource for the discovery of anti-MRSA natural medicines. In recent years, aqueous and ethanol extracts from various *Salvia* species have exhibited favorable inhibitory activity against MRSA [23], such as *S. albimaculata.* LC‑HRMS analysis indicated that salvianolic acid B and homoplantaginin are the main active components [24]. In addition, the essential oil of *S. sclarea* also shows strong anti‑MRSA activity [25]. However, the antibacterial potential of numerous Salvia species remains to be further investigated and exploited.

Multivariate analysis model can establish bioactive compounds correlation [10], and network pharmacology may predict potential targets interactions [11], which were used to explored anti-MRSA agents in *Salvia*. The combined strategy disclosed two unreported anti-MRSA compounds successfully, which can avoid repeating isolation of 7 plants routinely. Biofilm is a cohesive membrane layer formed by the excessive accumulation of a large number of bacteria surrounded by secreted polymers such as fibrin [26], which is difficult to eliminate and lack of target drug in market up to now [27]. Interestingly, miltirone and przewaquinone A showed scavenging effect on biofilm formation in a dose-dependent manner, respectively.

Phosphatidylglycerol is the main component of MRSA membrane [20], while carbohydrate metabolism is an important pathway for the energy required by bacteria [22]. Miltirone and przewaquinone A interfered with glycerolipid, glycolysis, gluconeogenesis, and other energy metabolic pathways, which could destroy membranes and inhibit their repair. Amino acids are fundamental units that make up proteins, and are crucial for bacterial growth [21]. Miltirone and przewaquinone A significantly influenced the metabolic pathway of various amino acids in MRSA by metabolomic analysis, which in turn might impair the synthesis and repair of proteins, as well as the production of carbon and nitrogen sources within bacteria, then affecting bacterial growth. The cell membrane serves as a vital protective barrier for cell, and its biophysical integrity and functionality are of paramount importance for bacterial growth and survival [28]. Miltirone and przewaquinone A could interfere with cell membrane function, resulting in the release of cellular contents and ultimately leading to the death of MRSA, respectively.

MRSA infections hinder wound healing [29], and bacterial colonization within wounds can reduce oxygen availability, thus preventing tissue regeneration. Then, effective management of bacterial infection is an essential prerequisite character for the successful treatment of wounds [30]. In our research, miltirone can improve wound healing in murine model, and concurrently mitigate bacterial colonization of wounds, showing roughly therapeutic effects as vancomycin.

In summary, two unreported anti-MRSA compounds from *Salvia* plants were revealed quickly and effectively using the combination of multivariate analysis and network pharmacology, and then their efficacy and pathway were validated by serial experiments in vitro and in vivo. The finding supported the traditional use of *Salvia* plants, and then presented miltirone and przewaquinone A as bactericide and biofilm scavenger, which should be attractive to chemists and biologists.

## Materials and methods

### Plant material

All air-dried plants were collected by the Kunming Zhifen Biotechnology Company from Yunnan Province in June 2020. Authentication of the plant material was also carried out by the company and checked at http://www.theplantlist.org. Voucher specimens have been deposited in the Plant Resource Library of the College of Pharmacy, Yunnan University.

### Strains, chemicals, reagents, and Galleria mellonella

The clinical isolates of MRSA003 were obtained from the First People's Hospital of Zunyi City. *Staphylococcus aureus* ATCC 25923 (MSSA), Multidrug-resistant (MDR) strains of *Enterococcus faecalis* (VRE, ATCC 51299), *Acinetobacter baumannii* (Bio-53272), and *Pseudomonas aeruginosa* (Bio-109004) were purchased from Beijing Bio-Bridge Technology Co., Ltd, China. MDR *Escherichia coli* (YIM B01141) was obtained from the Yunnan Institute of Microbiology. Mass-grade methanol, acetonitrile, isopropanol and formic acid were purchased from Merck. Watson’s water was purchased from Watsons Food and Beverage Co., Ltd. Dimethylsulfoxide (DMSO), TSA, TSB, PBS, vancomycin, ampicillin, cefoxitin, polymyxin B sulfate, polyformaldehyde, fluorescein isothiocyanate isomer (FITC), 4’,6-diamidino2-phenylindole (DAPI), isoamyl acetate, propidium Iodide (PI), 3,3’-dipropylthiadicarbocyanine iodide (DiSC3 [5]), and the Laurdan probe were purchased from Macklin Reagents (Shanghai, China). All standard compounds including przewaquinone A and miltirone (purity ≥ 98%) were obtained from Le Mei Tian (ChenDu, China). *Galleria mellonella* larvae were obtained from Huiyude Biotechnology Co., Ltd., China.

### Animals

Specific pathogenic-free female ICR mice (weight ranged from 16 to 23 g) were obtained from Kunming Medical University. All mice were raised in a laboratory of specific pathogen-free (SPF) level at room temperature (20–25℃) with a standard diet and water, constant humidity (40–70%), and a 12-h/12-h light–dark cycle. In accordance with international guidelines for animal experimentation and internationally recognized ethical principles for the use and care of experimental animals, the animal study was approved by the Institutional Animal Care and Use Committee of Yunnan University (animal ethics approval code: YNUCARE202210020).

### Preparation of extracts

The dried herbs were processed as described in the Supplementary Material (S1). Subsequently, all fractions were tested for anti-MRSA effect, resulting in the identification of nine bioactive fractions. Minimum inhibitory concentration (MIC) data were presented in Table S2, and the components yields were listed in Table S3.

### UPLC-Q-TOF–MS analysis and multivariate analysis

Chromatographic separation and mass spectral data of the fractions were obtained using ultra-high-performance liquid chromatography (UPLC) with a 6545 Q-TOF–MS system (Agilent Technologies, Palo Alto, CA, USA), and the instrument parameters were listed in the Supplementary Material (S2). Principal component analysis (PCA) was conducted using Origin software (Version 8.0) on 9 bioactive fractions from 7 plants to explore the correlation between *Salvia* extracts and anti-MRSA bioactivity.

### Network pharmacology

Network pharmacology analysis followed the literatures [11, 31] with a few modifications with details in the Supplementary Materia (S3).

### Antimicrobial bioactivity

#### Determination of the MIC and MBC

In accordance with the Clinical and Laboratory Standards Institute protocols (CLSI, 2021), the MIC and MBC of miltirone and przewaquinone A were determined using the broth microdilution method [32] in the Supplementary Material (S4).

#### Time–kill curve

The time-kill curves of miltirone and przewaquinone A against MRSA003 were analyzed as previously described [33] with details in Supplementary Material (S5).

#### Growth kinetics

Growth kinetic assay was used to assess MRSA003 growth and death [34], as described in the Supplementary Material (S6).

#### Antibiofilm assay

Quantitative analysis of the biofilms was performed using the crystal violet method, and biofilm observation was carried out using a fluorescence microscope following the literatures [3, 29], as described in Supplementary Material (S7).

#### Scanning electron microscope

Field-emission scanning electron microscopy (SEM; Nova Nano SEM 450, FEI, USA) was used to observe the effects of miltirone and przewaquinone A against MRSA003 [35] with details in Supplementary Material (S8).

#### Cell membrane integrity assay

The integrity of the MRSA003 membrane was evaluated using PI [28] in Supplementary Material (S9).

#### Cell membrane depolarization assay

3,3'-dipropylthiadicarbocyanine iodide (DiSC3 [5]) (Maokang Biotechnology Co., Ltd., Shanghai, China) was used to assess the membrane depolarization status of MRSA003 [36] with details in Supplementary Material (S10).

#### Evaluation of membrane fluidity

Membrane fluidity was evaluated with a Laurdan probe [28, 37] with details in Supplementary Material （S11）.

#### Nucleic acid leakage

The impact of miltirone and przewaquinone A on cell permeability were evaluated by quantifying the release of nucleic acids from cells into the medium [38] with detailed information in the Supplementary Material (S12).

#### *Cytotoxicity *in vivo

*G. mellonella* was used to evaluate the cytotoxicity of miltirone and przewaquinone A in vivo [38, 39], with the specific experimental procedure in Supplementary Material (S13).

#### Metabolomics analysis

MRSA003 was adjusted to a density of 1 × 10^7^ CFU/ml after culturing to the growth logarithmic phase and incubated for 4 h at 37 °C after treatment with or without 1/2 × MIC miltirone or przewaquinone A [40]. The MRSA003 metabolites were extracted and derivatized according to a previously described method [29], and detailed experimental procedures was provided in the Supplementary Material (S14).

#### *M*ice skin wound infection model

A mouse skin wound infection model was established to determine anti-MRSA bioactivity in vivo and the capacity to facilitate the wound healing process [29, 41], and the details were provided in Supplementary Material (S15).

### Data analysis

All assays were performed in triplicate and repeated three times independently. Data were analyzed by variance and the significance of the difference was evaluated with a Tukey’s test (*p* ≤ 0.05). Statistical analyses were performed using GraphPad Prism software (v.6.0; GraphPad Software, CA, USA). Multivariate statistical analyses of principal component analysis (PCA) and heatmaps were generated by Origin 2021 software (OriginLab, Northampton, MA, USA).

## Supplementary Information

Supplementary Material 1.

## Data Availability

The data supporting the results of this study can be obtained from the corresponding author on reasonable request.

## Abbreviations

CDC: Centers for Disease Control and Prevention
CFU: Colony-forming unit assays
DAPI: 4’,6-Diamidino-2-Phenylindole
FITC: Fluorescein isothiocyanate
MIC: Minimum inhibitory concentration
MBC: Minimum bactericidal concentration
PBS: Phosphate buffered solution
PCA: Principal component analysis
PPI: Protein–protein interaction
SEM: Scanning electron microscope
TIC: Total ion chromatograms
TSA: Tryptic soy agar
TSB: Tryptic soy broth
KEGG: Kyoto Encyclopedia of Genes and Genomes
GO: Gene Ontology
MT: Miltirone
PA: Przewaquinone A
