# Supplementary material for: Rapid exploration of anti-MRSA components from plants of Salvia
Source: Nat Prod Bioprospect. 2026 Jun 1;16(1):69. doi: 10.1007/s13659-026-00624-0 (PMC13226764; doi:10.1007/s13659-026-00624-0)
Supplement: Supplementary file 1 — Supplementary Material 1. [file 13659_2026_624_MOESM1_ESM.docx]

**Supporting Information**

**Rapid Exploration of Anti-MRSA Components from Plants of *Salvia***

Huan Huang^a,1^, Zhao-Jie Wang^a,1^, Li-Yu Bai^a^, Yue-Ming Jiang^a^, Yan-Yan Zhu^a^，Xiao-Dong Luo^a,b*^

*^a^Yunnan Characteristic Plant Extraction Laboratory, Key Laboratory of Medicinal Chemistry for Natural Resource, Ministry of Education and Yunnan Province, School of Chemical Science and Technology, Yunnan University, Kunming, 650500, People’s Republic of China*

*^b^State Key Laboratory of Phytochemistry and Plant Resources in West China, Kunming Institute of Botany, Chinese Academy of Sciences, Kunming 650201, People’s Republic of China*

__________________________________________________

* Corresponding author.

Tel: +86 0871 65032929;

*E-mail address:* xdluo@ynu.edu.cn (X.-D. Luo).

^1^ These authors contributed equally to this work.

**Contents**

**S1. Instrument parameters of UHPLC-Q-TOF-MS analysis.**

**S2. Experimental procedures in metabolomics analysis.**

**S3.** **Network pharmacology**

**S4. Determination of the MIC and MBC.**

**S5. Time–kill curve**

**S6. Growth kinetics assay**

**S7. Observation of biofilm**

**S8. Scanning electron microscope**

**S9. Cell membrane integrity assay**

**S10. Cell membrane depolarization assay**

**S11.** **Evaluation of membrane fluidity**

**S12. Nucleic acid leakage**

**S13. Cytotoxicity *in vivo***

**S14. Metabolomics analysis**

**S15. Mice skin wound infection model**

**Table S1. The specific information of seven *Saliva* herbs.**

**Table S2. MIC of bioactive fractions from *Salvia* plants.**

**Table S3. The ethanol extraction yields of seven plants and the yields of extractions subjected by food grade D101 macroporous resin column.**

**Table S4. Calibration curves, linear range, limit of detection (LOD), and limit of quantification (LOQ) of reference compounds.**

**Table S5. Determination of main compounds in bioactive fractions of *Salvias* by HPLC-Q-TOF-MS/MS (mg/g of dry extract).**

**Table S6. The MIC and MBC (μg/ml) results of MT and PA for Gram-positive and Gram-negative bacteria.**

**Table S7. The differences metabolites between the MT-treated group and the untreated group.**

**Table S8. The differences metabolites between the PA-treated group and the untreated group.**

**Fig.S1. The TIC of bioactive fractions in ESI+ (A) and ESI- (B) and the TIC of ethanol extracts (C) from seven plants of *Salvia.***

**Fig.S2. The structures of 44 compounds.**

**Fig.S3. MS^2^ spectra under multiple collision energies (A) and proposed fragmentation pathway (B) of przewaquinone A (15)**

**Fig.S4. A: Protein-protein interaction network. B: GO function annotation.**

**Fig.S5. A: Docking simulation of MT with DNA and DNA gyrase. B: Docking simulation of PA with DNA and DNA gyrase.**

**S1. Instrument parameters of UHPLC-Q-TOF-MS analysis.**

All samples were dissolved in DMSO and diluted to 100 µg/ml with methanol. Agilent SB C18 (2.1 mm × 100 mm, 1.8 µm, Agilent Technologies) was used to separate. The mobile phase consists of 0.1% formic acid water (phase A) and pure acetonitrile (B) and the injection volume is 2 μL. The elution procedure is 0 − 1 min, 5-15% B; 1 − 3 min, 15 − 60% B; 3 − 8 min, 60-80% B; 8 − 15 min, 80 − 98% B; 15 − 18 min, 98% B. MS spectra were acquired in positive and negative ion mode (ESI). The gas temperature was 325°C, the dying gas flow rate was 9 L/min, the nebulizer pressure was 35 psi, the sheath gas temperature was 365 °C with a flow rate of 11 L/min, and the fragmentor was 120 V. The OctopoleRFPeak was 750 V, and the skimmer voltage was 65 V. The nozzle voltage was 0.5 kV and the VCap voltage was 3.5 kV with an ESI source in positive ion mode. The nozzle voltage was 1.5 kV and the VCap voltage was 3 kV with an ESI source in negative ion mode. Using full scan mode to collect MS1 data, with a quality collection range of m/z 20-2000 and a collection rate 1 spectrum/s. Using auto-MS2 mode to simultaneously collect MS1 and MS2 data. The data quality collection ranges of MS1 and MS2 are m/z 100-1000, and the collection rates are 3 and 2 spectra/s, respectively. The MS/MS fragments of the typical substances were acquired by collision energies optimized from 10 to 40 eV.

**S2. Experimental procedures in metabolomics analysis.**

UHPLC-Q-TOF-MS was used for data collection and BEH Amide HILIC normal phase chromatography column (2.1 × 150 mm, 1.7 µm, ACQUITY UPLC^®^ BEH Amide, Waters) was used to separate. Inject with an automatic sampler, with a sample volume of 2 μl while the temperature of the chromatographic column is 38°C. Gradient elution was performed using 0.1% formic acid water (phase A) and pure acetonitrile (phase B) as flowability, with a mobile phase flow rate of 0.15 ml/min. The elution procedure is 0 − 1 min, 95% B; 1 − 16 min, 95 − 70% B; 17 − 22 min, 70 − 30% B; 23 − 28 min, 30% B; 28 − 30 min, 30-95% B. Other instrument parameters are recorded in the supporting information. Preliminary processing of raw data using MSDIAL, annotation of metabolites using databases such as Metlin ([https://metlin.scripps.edu](https://metlin.scripps.edu/)), MassBank (<https://massbank.eu/>), MoNA ([mona.fiehnlab.ucdavis.edu](http://mona.fiehnlab.ucdavis.edu)), and HMDB ([https://hmdb.ca](https://hmdb.ca/)), and multivariate analysis using R package ([www.metaboanalyst.ca](http://www.metaboanalyst.ca)).

**S3. Network pharmacology**

Utilizing TCMSP (https://old.tcmsp-e.com/tcmsp.php), PubChem (https://pubchem.ncbi.nlm.nih.gov), and Swiss target prediction websites search to (http://www.swisstargetprediction.ch/) predict the targets of the main components of biological fractions. The targets of anti-MRSA were obtained from GeneCards (https://www.genecards.org/), UniProt (https://www.uniprot.org/) and NIBI PubMed (https://www.ncbi.nlm.nih.gov/assembly/). The Jvenn was used to filter intersection targets from the main components of biological fractions and anti-MRSA which were taken for the potential targets (https://www.bioinformatics.com.cn). After that, the protein-protein interaction (PPI) network and “disease targeted drug target” network of intersections was constructed by STRING database (https://cn.string-db.org) and visualized with Cytoscape 3.7.1 (Cytoscape Consortium, CA, USA). The Gene Ontology (GO) and Kyoto Encyclopedia of Genes and Genomes (KEGG) analysis were constructed by DAVID database (https://david.ncifcrf.gov/). The results were visualized with Micro Bioinformatics - Online Bioinformatics Analysis and Visualization Cloud Platform (<https://www.bioinformatics.com.cn>).

**S4. Determination of the MIC and MBC.**

Bacteria were cultivated to the logarithmic growth phase, and adjusted bacterial density to 1 × 10^5^ CFU/ml. The MT and PA were continuously twofold serial dilution in a 96-well microliter plate and the range is between 128 to 2 μg/ml. MIC and MBC were assessed after incubation at 37°C for 24 hours.

**S5. Time–kill curve**

MRSA003 was cultivated to logarithmic phase (1 × 105 CFU/ml) and incubated with MT and PA (1, 2 and 4 × MIC), vancomycin (1 × MIC) and solvent (equal volume) at 37°C for 0, 1, 2, 4, 6, 8, 12, 24, 36, and 48 hours. After continuous dilution with PBS, each sample was spread on the TSA agar. The colony count was calculated after incubation at each time point and the values (Log10 CFU/ml) were obtained.

**S6. Growth kinetics assay**

The bacterial suspension (1 × 10^7^ CFU/ml) was treated with MT and PA at 1/2, 1, 2 and 4 × MIC; the blank control group was untreated, while the positive control groups were treated with vancomycin at 1 and 2 × MIC and then incubated at 37°C. To assess the growth, optical density at 600 nm (OD_600_) was measured at 0, 30, 60, 120, 240, 360, 540, 720, 1320, 1440, and 1800 min by Enzyme-Linked Immunosorbent, respectively.

**S7. Observation of biofilm**

500 μl of MRSA003 solution (1 × 10^7^ CFU/ml) was added into 24-well plate with a coverslip and incubated for 24 hours to form biofilm. It was sucking up planktonic bacteria and washing with PBS two times after biofilm formation. The treatment methods are the same as in the previous step. After sucking out the upper liquid, the biofilm was fixed with 4% (v/v) paraformaldehyde at room temperature for 1 hour and washed with PBS three times. Biofilm was stained with 10 μg/ml fluorescein isothiocyanate (FITC) and 20 μg/mL 4’,6-Diamidino-2-Phenylindole (DAPI) at room temperature in the dark for 1 hour, respectively. Then, FITC and DAPI were sucked out from 24 wells; biofilm was washed by H_2_O again. Lastly, the cover-slip was removed and placed on the slide, then sealed and observed under the fluorescence microscope.

**S8. Scanning electron microscope**

3 ml MRSA003 suspension (OD600 = 0.2) was seeded into centrifuge tubes. The experimental group was treated with 4 × MIC MT or PA; the blank control group was treated with DMSO; the positive control group was treated with 4 × MIC vancomycin and incubated at 37°C for 4 hours. Then, 4% paraformaldehyde was added into tubes for 24 hours at 4°C and dehydrated by 30%, 50%, 70%, 90%, 100% ethanol (v/v) and 50%, 100% isoamyl acetate (v/v) for 10 min successively. After that, the bacteria were removed from centrifuge tubes, which dried with CO2 and sprayed with gold by cathodic and used for SEM.

**S9. Cell membrane integrity assay**

Overnight cultures of MRSA003 to logarithmic phase, centrifuged and resuspended with PBS to adjust bacterial suspensions to approximately an OD600 of 0.5, followed by the addition of MT or PA at 1/2, 1, and 2 × MIC, and the positive control group was treated with 1% TritonX-100; the blank control group was treated with PBS. PI was added to the bacterial solution with a final concentration of 10 μm. After incubation at 37°C for 1 hour in the dark, the fluorescence intensity was measured at an excitation wavelength of 535 nm and an emission wavelength of 615 nm.

**S10.** **Cell membrane depolarization assay**

Overnight cultures of MRSA003 to logarithmic phase, centrifuged and re-suspended with PBS to adjust bacterial suspensions to approximately an OD600 of 0.2. The bacterial suspension was aspirated into a black 96-well cell culture plate, and the final concentration of 10 μm DISC3 (5) was added, followed by incubation for 1 hour in the dark. The fluorescence intensity of the mixture was measured continuously for 15 min at the excitation wavelength of 620 nm and the emission wavelength of 670 nm, every 3 min. After the fluorescence intensity was stabilized, MT and PA at 1 and 2 × MIC were added and the blank control group was treated with PBS. After that, the fluorescence intensity was continued to be measured for 45 min, every 3 min.

**S11. Evaluation of membrane fluidity**

MRSA003 was cultivated to the logarithmic phase and centrifuged and re-suspended with PBS to adjust bacterial suspensions to approximately an OD_600_ nm of 0.2. Laurdan with a final concentration of 10 μm mixed with bacterial suspensions followed by incubation at 37°C for 10 min in the dark. Subsequently, MT and PA with 1 and 2 × MIC were added in bacterial suspensions and incubated at 37°C for 35 min. After that, the fluorescence was determined with Ex/Em = 350/440 or 350/490 nm. Laurdan generalized polarization (GP) was calculated by the following formula: Laurdan GP = (I_440_-I_490_)/(I_440_+I_490_).

**S12.** **Nucleic acid leakage**

Overnight cultures of MRSA003 to logarithmic phase, centrifuged and resuspended with stroke-physiological saline solution to adjust bacterial suspensions to approximately an OD600 nm of 0.3. Bacterial suspensions were mixed with MT and PA in 1 and 2 × MIC followed by incubation at 37°C for 1 and 2 hours in the dark. Next, bacteria were centrifuged and the measured absorbance of the supernatant was at 260 nm.

**S13.** **Cytotoxicity *in vivo***

All *G. mellonella* larvae were divided into 10 groups at random. Then, the *G. mellonella* larvae were treated with MT and PA (2 mg/kg, 4 mg/kg, 8 mg/kg, 16 mg/kg, 32 mg/kg) on the right posterior gastropod and the blank control group was treated with PBS. The survival rate of *G. mellonella* larva within 7 days was recorded.

**S14.** **Metabolomics** **analysis**

UHPLC-Q-TOF-MS was used for data collection and BEH Amide HILIC normal phase chromatography column (2.1 × 150 mm, 1.7 µm, ACQUITY UPLC® BEH Amide, Waters) was used to separate. Inject with an automatic sampler, with a sample volume of 2 μl while the temperature of the chromatographic column is 38°C. Gradient elution was performed using 0.1% formic acid water (phase A) and pure acetonitrile (phase B) as flowability, with a mobile phase flow rate of 0.15 ml/min. The elution procedure is 0 − 1 min, 95% B; 1 − 16 min, 95 − 70% B; 17 − 22 min, 70 − 30% B; 23 − 28 min, 30% B; 28 − 30 min, 30-95% B. Other instrument parameters are recorded in the supporting information. Preliminary processing of raw data using MSDIAL, annotation of metabolites using databases such as Metlin (https://metlin.scripps.edu), MassBank (https://massbank.eu/), MoNA (mona.fiehnlab.ucdavis.edu), and HMDB (https://hmdb.ca), and multivariate analysis using R package ([www.metaboanalyst.ca](http://www.metaboanalyst.ca)).

**S15.** **Mice skin wound infection model**

The research was conducted in accordance with the internationally accepted principles for laboratory animal use and care. Healthy female Kunming mice (28 – 32 g) were used for the skin wound infection model. After one week of adaptation in the animal facility, randomly divided into five groups (n = 5). After three days of cyclophosphamide treatment, mice were anesthetized by pentobarbital sodium solution and depilated in the back. Wounds (6 mm × 6 mm) on the back were achieved using surgical forceps and injected with 50 μl MRSA003 solution (2 × 109 CFU/ml). Four hours later, the positive drug group was treated with vancomycin (4 mg/kg); two experimental groups were treated with high and low doses of MT (4 mg/kg and 2 mg/kg), the model group was treated with PBS and the blank group was no injection of MRSA003 and compound. After 7 days of continuous administration, all mice were euthanized and the infected skin was excised, homogenized in sterile PBS and counted with TSA board. Furthermore, the skins were stained with hematoxylin and eosin (H&E) to observe their pathological changes under a microscope.

T**able 1.** The specific information of seven *Saliva* herbs.

| abbreviation | SsM | | SpG | | SmL | | | StF | | | Scft | | SpH | | SpM | |
| --- | --- | --- | --- | --- | --- | --- | --- | --- | --- | --- | --- | --- | --- | --- | --- | --- |
| Plant resources | 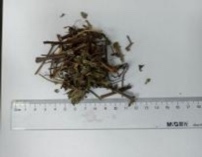 | | 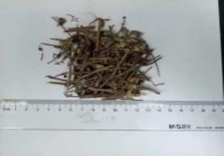 | | 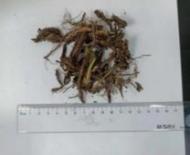 | | | 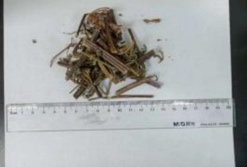 | 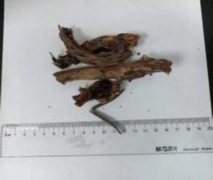 | | | | 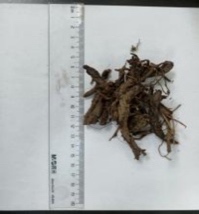 | | 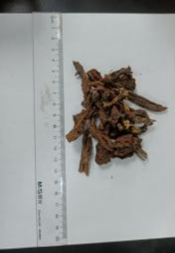 | |
| Scientific name | *Salvia sinica* Migo. | | *Salvia plectranthoides* Griff. | | *Salvia mairei* Levl. | | | *Salvia tricuspis* Franch. | *Salvia castanea* f. tomentosa. | | | | *Salvia prattii* Hemsl. | | *Salvia przewalskii* Maxim. | |
| Medicinal part | Whole plant | | Whole plant | | Whole plant | Whole plant | | | Whole plant | | | | Whole plant | | Whole plant | |
| No | | Xdl-pl-253 | | Xdl-pl-254 | Xdl-pl-255 | | Xdl-pl-256 | | Xdl-pl-260 | | | Xdl-pl-262 | | Xdl-pl-263 | |  |
| Harvest place | Hubei, China | | Hubei, China | | Yunnan, China | Sichuan, China | | | | | Xizang, China | | Sichuan, China | | Xizang, China | |
| Tradition-al use | Cooling blood and resolving carbuncle | | resolving carbuncle | | – | metrorrhagia, haematemesis, and traumatic haemorrhage | | | | Detumescence, blood-stasis-removing | | | resolving carbuncle | | Cooling blood and resolving carbuncle | |
| References | [1, 2] | | [3, 4] | | – | [5] | | | | | [6] | | [3] | | [2, 7] | |

**Table S2**. MIC of bioactive fractions from *Salvia* plants.

| Fractions | MRSA003 (MIC μg/ml) | |
| --- | --- | --- |
| SsM-80% | 256 | |
| SpG-80% | 256 |  |
| SmL-80% | 32 |  |
| StF-80% | 128 |  |
| StF-95% | 256 |  |
| Scft-80% | 256 |  |
| SpH-80% | 64 |  |
| SpM-80% | 32 |  |
| SpM-95% | 16 |  |
| Vancomycin | 2 |  |
| Ampicillin | 512 |  |

**Table S3.** The ethanol extraction yields of seven plants and the yields of extractions subjected by food grade D101 macroporous resin column.

Note: data are expressed as percentages.

|  | SsM | SpG | SmL | StF | Scft | SpH | SpM |
| --- | --- | --- | --- | --- | --- | --- | --- |
| extraction rate | 9.88 | 10.38 | 6.77 | 14.65 | 6.54 | 5.39 | 8.68 |
| water | 35.98 | 40.48 | 17.93 | 24.69 | 41.65 | 20.98 | 36.05 |
| 20% ethanol | 7.23 | 14.36 | 11.18 | 5.97 | 8.06 | 9.48 | 0.99 |
| 40% ethanol | 6.55 | 14.45 | 16.26 | 4.41 | 6.07 | 9.98 | 7.15 |
| 60% ethanol | 10.37 | 4.34 | 4.16 | 2.99 | 9.75 | 5.57 | 3.65 |
| 80% ethanol | 5.79 | 5.66 | 20.20 | 3.34 | 6.40 | 13.06 | 13.19 |
| 95% ethanol | 5.31 | 11.09 | 17.66 | 2.92 | 8.56 | 20.09 | 19.78 |

**Table S4.** Calibration curves, linear range, limit of detection (LOD), and limit of quantification (LOQ) of reference compounds.

| Compounds | Regression equation | Correlation coefficient (R^2^) | Linear range (μg/μL) | LOD (μg/μL) | LOQ (μg/μL) |
| --- | --- | --- | --- | --- | --- |
| Dihydrotanshinone I (ESI+) | y = 2E-06x - 0.185 | 0.9948 | 0.04-10.24 | 0.02 | 0.03 |
| Kumatakenin (ESI+) | y =9E-07x - 0.1993 | 0.994 | 0.04-10.24 | 0.02 | 0.04 |
| Kumatakenin (ESI-) | y = 0.0006x - 0.024 | 0.9994 | 0.001-0.016 | 0.0005 | 0.0008 |
| Ursolic acid (ESI+) | y = 4E-06x - 0.1584 | 0.9966 | 0.08-10.24 | 0.03 | 0.04 |
| Ursolic acid (ESI-) | y = 6E-07x + 1.1074 | 0.9908 | 0.00025-0.016 | 0.0001 | 0.0002 |
| Cryptotanshinone (ESI+) | y = 2E-07x - 0.1726 | 0.9977 | 0.02-10.24 | 0.005 | 0.02 |


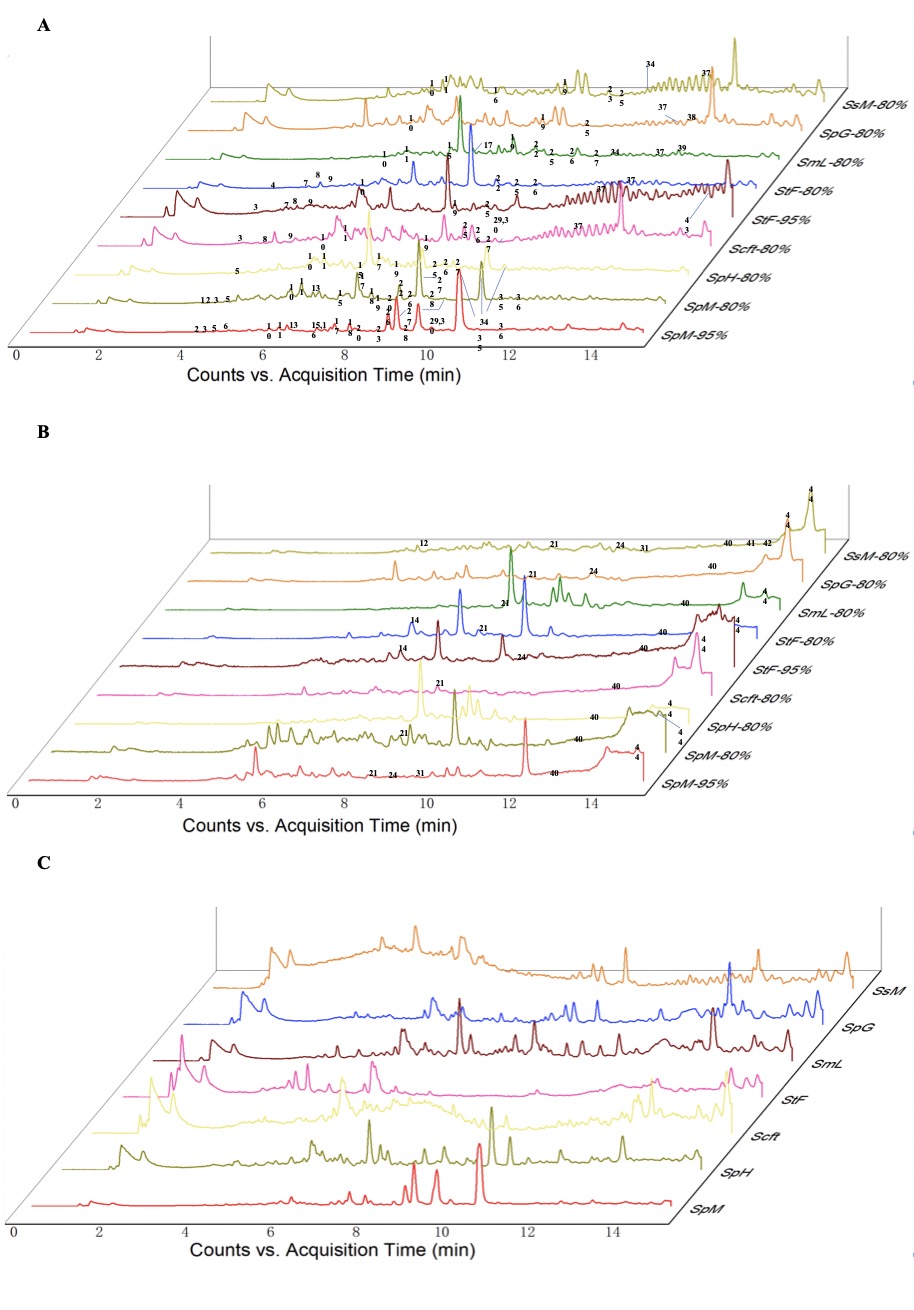


**Fig.S1.** The TIC of bioactive fractions in ESI+ (A) and ESI- (B) and the TIC of ethanol extracts (C) from seven plants of *Salvia.*

**Fig.S2.** The structure of 44 compounds.

**Fig.S3.** MS^2^ spectra under multiple collision energies (A) and proposed fragmentation pathway (B) of przewaquinone A (15)

**Table S5.** Determination of main compounds in bioactive fractions of *Salvias* by HPLC-Q-TOF-MS/MS (mg/g of dry extract).

| Compounds | SsM-80% | SpG-80% | SmL-80% | StF-80% | StF-95% | Scft-80% | SpH-80% | SpM-80% | SpM-95% |
| --- | --- | --- | --- | --- | --- | --- | --- | --- | --- |
| Kumatakenin **(1)**^2^ | － | － | － | － | － | － | － | 0.01±0 | － |
| Tanshindiol B **(**2)^1^ | － | － | － | － | － | － | － | 1.27±0.06^a^ | 2.29±0.08^b^ |
| 5,6,4’-trihydroxy-7,3’-dimethoxyflavone **(4)**^2^ | － | － | － | 0.17±0.005 | － | － | － | － | － |
| Tanshindiol C **(5)**^1^ | － | － | － | － | － | － | 0.032±0^a^ | 1.11±0.16^b^ | 2.47±0.18^b^ |
| Diosmetin **(7)**^2^ | － | － | － | 1.12±0.05^a^ | 0.08±0.02^b^ | － | － | － | － |
| Velutin **(8)**^2^ | － | － | － | 1.14±0.05^a^ | 0.22±0^b^ | 0.03±0^c^ | － | － | － |
| Genkwanin **(9)**^2^ | － | － | － | 5.23±0.11^a^ | 6.3±0.01^a^ | 0.03±0^b^ | － | － | － |
| Angoletin **(10)**^2^ | 0.23±0.01^a^ | 0.008±0^b^ | 0.57±0.02^a^ | 0.004±0^b^ | － | 0.5±0^a^ | 9.29±0.02^c^ | 0.66±0.01^a^ | 0.065±0.1^d^ |
| 7-(Acetyloxy)-3-phenyl-6-propyl-4H-1-benzopyran-4-one **(11)**^2^ | 0.056±0.01^b^ | － | 0.17±0.02^a^ | － | － | 0.17±0.04^a^ | 1.8±0.01^c^ | 2.18±0.1^c^ | 0.34±0.01^a^ |
| Rosmanol **(12)** | 0.006±0 | － | － | － | － | － | － | － | － |
| Trijuganone C **(13)**^1^ | － | － | － | － | － | － | － | 2.09±0.07^a^ | 0.75±0.07^b^ |
| Acacetin **(14)**^2^ | － | － | － | 2.66±0.13 | 3.63±0.1 | － | － | － | － |
| Przewaquinone A **(15)**^3^ | － | － | 0.41±0.05^b^ | － | － | － | 1.09±0.05^ab^ | 2.54±0.1^a^ | 4.17±0.06^a^ |
| Neocryptotanshinone **(17)**^1^ | － | － | 0.82±0.06^b^ | － | － | － | 1.41±0.05^ab^ | 11.17±0.5^c^ | 3.49±0.11^a^ |
| Methyl tanshinonate **(18)**^1^ | － | － | － | － | － | － | － | 8.72±1^a^ | 25.43±0.9^b^ |
| Ceanothic acid **(19)**^4^ | 2.13±0.08^a^ | 0.97±0.02^b^ | 6.07±0.28^c^ | － | 7.34±0.13^c^ | 5.23±0.27^cd^ | 4.76±0.47^d^ | 2.13±0.08^a^ | － |
| Danshenxinkun B **(20)**^1^ | － | － | － | － | － | － | － | 8.67±1 | 10.39±0.08 |
| Asiatic acid **(21)**^4^ | 5.98±0.02 | 5.98±0.018 | 5.99±0.01 | 5.91±0.11 | － | 6.46±0.03 | － | 6.06±0.26 | 5.87±0.45 |
| Glycyrrhetic acid **(25)**^4^ | 8.7±0.36^a^ | 12.8±0.54^b^ | 5.15±0.15^c^ | 0.7±0.01^d^ | 0.22±0.09^d^ | 13.34±0.06b | 8.7±0.36^a^ | － | － |
| Tanshinone I **(26)**^1^ | － | － | 0.13±0^b^ | 0.01±0^a^ | － | 0.78±0.02^c^ | 0.01±0^a^ | 14.68±1.3^d^ | 90.37±6.8e |
| Cryptotanshinone **(27)^3^** | － | － | 0.66±0.05^a^ | － | － | 0.021±0^b^ | 5.2±0^c^ | 28.15±0.64^d^ | 23.7±0.07^d^ |
| Bavachin **(28)**^2^ | － | － | － | － | － | － | － | 0.004±0^a^ | 2.08±0.1^b^ |
| 1,2-Dihydrotanshinquinone **(29)**^1^ | － | － | － | － | － | 0.026±0.01^a^ | － | 3.84±0.34^b^ | 107.56±0.2^c^ |
| Dihydrotanshinone I **(30)**^1^ | － | － | － | － | － | 0.026±0^a^ | － | 3.64±0^b^ | 108.56±0.03^c^ |
| Glycyrrhetinic acid **(32)**^4^ | 0.35±0.01^a^ | － | 8.8±0.4^b^ | － | － | 1.84±0^c^ | 4.06±0.3^d^ | 2.86±0.46^c^ | 0.35±0.01^a^ |
| Maslinic acid **(33)**^4^ | 6.27±0.08 | 5.99±0.34 | 6.26±0.02 | 6.04±0.12 | 5.65±0.09 | 6.14±0.05 | 6±0.06 | 6±0.12 | 5.57±0.02 |
| Tanshinone ⅡA **(34)**^1^ | 0.1±1^a^ | － | 0.21±0.01^b^ | － | － | － | 0.89±0.01^c^ | 15.5±1.02^d^ | 61.8±1.4^e^ |
| Miltirone **(35)**^1^ | － | － | － | － | － | － | － | 0.94±0^a^ | 3.97±0.05^b^ |
| Trenbolone **(36)**^1^ | － | － | － | － | － | － | － | 0.44±0.07^a^ | 2.06±0.8^b^ |
| Oleanolic acid **(37)**^4^ | 4.56±0.43^ad^ | 5.07±0.05^a^ | 0.72±0.04 ^b^ | 1±0.07^c^ | 1.07±0.15^c^ | 3.85±0.1^d^ | － | － | － |
| Arjungenin **(38)**^4^ | － | 0.04±0 | － | － | － | － | － | － | － |
| Ziyuglycoside II **(39)**^4^ | － | － | 0.8±0 | － | － | － | － | － | － |
| 18α-Glycyrrhetinic acid **(40)**^4^ | 5.6±0.12 | 5.67±0.12 | 5.72±0.01 | 5.56±0.05 | 5.56±0.04 | 5.6±0.03 | 5.55±0.03 | 5.57±0.04 | 5.4±0.2 |
| Hederagenin **(41)**^4^ | 5.55±0.12 | － | － | － | － | － | － | － | － |
| Saikogenin D **(43)**^4^ | － | － | － | － | 3.87±0.08 | － | － | － | － |
| Ursolic acid **(44)**^4^ | 15.56±1.44^a^ | 15.55±1.4^a^ | 9.29±0.13^b^ | 7.02±0.23^cd^ | 8.24±0.19^bd^ | 14.16±0.07^a^ | 6.24±0.07^c^ | 6.07±0.1^c^ | 5.8±0.2^c^ |

The numerical value is expressed as the mean ±SD of three parallel experiments; The means are statistically significant which continuous use of the same letter indicates in a raw; “－” means not detected. 1: This compound was semi-quantified by Dihydrotanshinone I; 2: This compound was semi-quantified by Kumatakenin; 3: This compound was semi-quantified by Cryptotanshinone; 4: This compound was semi-quantified by Ursolic acid.

**Fig.S4.** A: Protein-protein interaction network. B: GO function annotation.

**Table S6.** The MIC and MBC (μg/ml) results of MT and PA for Gram-positive and Gram-negative bacteria.

| Organism | | **Gram-Positive** | | | **Gram-Negative** | | |
| --- | --- | --- | --- | --- | --- | --- | --- |
|  |  | *S.aureus* ATCC 25923 | MRSA | VRE | *A.baumanii* Bio-53272 (MDR) | *P.aeruginosa* Bio-10904 (MDR) | *E. Coli* YIM B01141 (MDR) |
| MT | MIC | 2 | 4 | 16 | >128 | >128 | >128 |
|  | MBC | 2 | 4 | >128 | - | - | - |
| PA | MIC | 4 | 8 | 16 | >128 | >128 | >128 |
|  | MBC | 32 | 32 | >128 | - | - | - |
| Neocryptotanshinone | MIC | 64 | 128 | >128 | >128 | >128 | >128 |
|  | MBC | - | - | - | - | - | - |
| Vancomycin | MIC | 0.5 |  | >128 | >128 | - | - |
| Ampicillin | MIC | <0.25 |  | 2 | >128 | >128 | 16 |
| Polymyxin B sulfate | MIC | - | - | - | 1 | 0.5 | 0.5 |

**Table S7.** The differences metabolites between the MT-treated group and the untreated group.

| No. | Average.Rt(min) | Average.Mz | Metabolite.name | FC | VIP | Adduct.type | Formula |
| --- | --- | --- | --- | --- | --- | --- | --- |
| 1 | 21.849 | 342.16809 | Naltrexone | 23253 | 1.6774 | [M+H]^+^ | C_20_H_23_NO_4_ |
| 2 | 13.336 | 313.12946 | Ranitidine | 15591 | 1.6018 | [M-H]^-^ | C_13_H_22_N_4_O_3_S |
| 3 | 2.745 | 283.17072 | Miltirone | 5333.1 | 1.768 | [M+H]^+^ | C_19_H_22_O_2_ |
| 4 | 3.471 | 331.15805 | Gibberellin A4 | 3188 | 1.8138 | [M-H]^-^ | C_19_H_24_O_5_ |
| 5 | 2.747 | 297.15005 | Cryptotanshinone | 1622.3 | 1.7716 | [M+H]^+^ | C_19_H_20_O_3_ |
| 6 | 3.653 | 452.18649 | Doxazosin | 1080.6 | 1.79 | [M+H]^+^ | C_23_H_25_N_5_O_5_ |
| 7 | 3.481 | 287.16751 | 2-Hydroxyestradiol | 816.08 | 1.8154 | [M-H]^-^ | C_18_H_24_O_3_ |
| 8 | 3.448 | 356.18707 | Glaucine | 727.47 | 1.7945 | [M+H]^+^ | C_21_H_25_NO_4_ |
| 9 | 3.677 | 313.14532 | Olanzapine | 610.32 | 1.8102 | [M+H]^+^ | C_17_H_20_N_4_S |
| 10 | 3.226 | 370.20328 | Corydaline | 512.24 | 1.7778 | [M+H]^+^ | C_22_H_27_NO_4_ |
| 11 | 3.287 | 303.16019 | Nordihydroguaiaretic Acid | 414.99 | 1.7722 | [M+H]^+^ | C_18_H_22_O_4_ |
| 12 | 10.604 | 431.25699 | Trandolapril | 370.6 | 1.731 | [M+H]^+^ | C_24_H_34_N_2_O_5_ |
| 13 | 2.823 | 299.16711 | 4-Androsten-3,17-dione 19-aldehyde | 365.31 | 1.7471 | [M-H]^-^ | C_19_H_24_O_3_ |
| 14 | 5.131 | 438.2388 | Lunarine | 295.77 | 1.7672 | [M+H]^+^ | C_25_H_31_N_3_O_4_ |
| 15 | 3.364 | 338.17563 | Olopatadine | 293.92 | 1.7745 | [M+H]^+^ | C_21_H_23_NO_3_ |
| 16 | 11.825 | 852.32568 | Paclitaxel | 265.81 | 1.7919 | [M-H]- | C_47_H_51_NO_14_ |
| 17 | 2.848 | 627.29474 | Gambogic acid | 264.45 | 1.781 | [M-H]^-^ | C_38_H_44_O_8_ |
| 18 | 9.432 | 719.24902 | Azadirachtin A | 251.05 | 1.7801 | [M-H]^-^ | C_35_H_44_O_16_ |
| 19 | 3.209 | 300.17813 | Intermedine | 246.16 | 1.7671 | [M+H]^+^ | C_15_H_25_NO_5_ |
| 20 | 2.764 | 313.14456 | BENZYLBUTYLPHTHALATE | 223.46 | 1.7335 | [M+H]^+^ | C_19_H_20_O_4_ |
| 21 | 3.679 | 345.12854 | cynaropicrin | 202.41 | 1.8111 | [M-H]- | C_19_H_22_O_6_ |
| 22 | 3.739 | 437.24609 | HC Toxin | 196.85 | 1.7883 | [M+H]^+^ | C_21_H_32_N_4_O_6_ |
| 23 | 2.664 | 597.30774 | Salannin | 194.99 | 1.7642 | [M+H]^+^ | C_34_H_44_O_9_ |
| 24 | 2.437 | 391.12421 | Shanzhiside | 160.88 | 1.7686 | [M-H]- | C_16_H_24_O_11_ |
| 25 | 22.098 | 314.13684 | Kresoxim-methyl | 151.87 | 1.7766 | [M+H]^+^ | C_18_H_19_NO_4_ |
| 26 | 22.465 | 386.25262 | Buspirone | 143.83 | 1.6248 | [M+H]^+^ | C_21_H_31_N_5_O_2_ |
| 27 | 23.947 | 417.18365 | Cyproterone acetate | 115.24 | 1.7356 | [M+H]^+^ | C_24_H_29_ClO_4_ |
| 28 | 3.593 | 369.2182 | Hirsutine | 113.41 | 1.7941 | [M+H]^+^ | C_22_H_28_N_2_O_3_ |
| 29 | 22.33 | 226.15619 | PROMETON | 105.56 | 1.7663 | [M+H]^+^ | C_10_H_19_N_5_O |
| 30 | 2.848 | 368.21338 | Propantheline | 96.886 | 1.7742 | [M+H]^+^ | C_23_H_29_NO_3_ |
| 31 | 6.826 | 419.27249 | Simvastatin | 96.413 | 1.737 | [M+H]^+^ | C_25_H_38_O_5_ |
| 32 | 25.247 | 299.20938 | Geissoschizoline | 79.936 | 1.5243 | [M+H]^+^ | C_19_H_26_N_2_O |
| 33 | 3.082 | 358.20316 | Laudanosine | 77.926 | 1.7538 | [M+H]^+^ | C_21_H_27_NO_4_ |
| 34 | 22.266 | 198.12511 | 2-(3-Phenylpropyl)pyridine | 73.045 | 1.7925 | [M+H]^+^ | C_14_H_15_N |
| 35 | 22.454 | 331.23029 | Deoxycorticosterone | 72.736 | 1.7723 | [M+H]^+^ | C_21_H_30_O_3_ |
| 36 | 2.783 | 442.23895 | HT-2 Toxin | 66.911 | 1.7576 | [M+H]^+^ | C_22_H_32_O_8_ |
| 37 | 24.961 | 399.14734 | S-Adenosylmethionine | 66.142 | 1.8022 | [M+H]^+^ | C_15_H_22_N_6_O_5_S |
| 38 | 24.722 | 412.04443 | Carfentrazone-ethyl | 66.04 | 1.8001 | [M+H]^+^ | C_15_H_14_C_l2_F_3_N_3_O_3_ |
| 39 | 22.418 | 453.24564 | Diphenoxylate | 65.347 | 1.7305 | [M+H]^+^ | C_30_H_32_N_2_O_2_ |
| 40 | 2.75 | 674.3714 | Substance P | 62.73 | 1.726 | [M+H]^+^ | C_63_H_98_N_18_O_13_S |
| 41 | 3.733 | 388.16083 | Flurazepam | 59.865 | 1.7419 | [M+H]^+^ | C_21_H_23_ClFN_3_O |
| 42 | 24.369 | 298.09863 | 5'-Methylthioadenosine | 58.252 | 1.5355 | [M+H]^+^ | C_11_H_15_N_5_O_3_S |
| 43 | 15.838 | 313.14423 | Clomipramine | 50.946 | 1.8003 | [M-H]- | C_19_H_23_ClN_2_ |
| 44 | 9.751 | 415.23013 | Tentoxin | 49.283 | 1.7749 | [M+H]^+^ | C_22_H_30_N_4_O_4_ |
| 45 | 3.562 | 975.38104 | Crocin | 48.018 | 1.7153 | [M-H]- | C_44_H_64_O_24_ |
| 46 | 22.462 | 216.13362 | Cycloate | 47.076 | 1.8044 | [M+H]^+^ | C_11_H_21_NOS |
| 47 | 22.273 | 513.28082 | Ganoderenic acid D | 44.714 | 1.7189 | [M+H]^+^ | C_30_H_40_O_7_ |
| 48 | 2.398 | 461.0387 | Kaempferol 3-glucuronide | 44.546 | 1.7736 | [M-H]- | C_21_H_18_O_12_ |
| 49 | 22.267 | 175.10782 | L-Theanine | 42.914 | 1.756 | [M+H]^+^ | C_7_H_14_N_2_O_3_ |
| 50 | 22.406 | 287.13882 | Scutellarein | 41.129 | 1.7298 | [M+H]^+^ | C_15_H_10_O_6_ |
| 51 | 13.258 | 272.98257 | ferulic acid 4-sulfate | 37.699 | 1.6712 | [M-H]- | C_10_H_10_O_7_S |
| 52 | 3.2 | 345.2085 | OXYPHENCYCLIMINE | 36.369 | 1.7272 | [M+H]^+^ | C_20_H_28_N_2_O_3_ |
| 53 | 2.896 | 399.1424 | aschantin | 34.867 | 1.7998 | [M-H]- | C_22_H_24_O_7_ |
| 54 | 22.501 | 175.12137 | N-Methyltryptamine | 34.281 | 1.7136 | [M+H]^+^ | C_11_H_14_N_2_ |
| 55 | 22.421 | 180.09927 | Fusaric acid | 33.812 | 1.6977 | [M+H]^+^ | C_10_H_13_NO_2_ |
| 56 | 5.874 | 210.04355 | Dimethirimol | 32.775 | 1.6984 | [M+H]^+^ | C_11_H_19_N_3_O |
| 57 | 24.937 | 367.19302 | Hirsuteine | 32.702 | 1.7147 | [M+H]^+^ | C_22_H_26_N_2_O_3_ |
| 58 | 22.365 | 326.12097 | Monocrotaline | 32.126 | 1.7165 | [M+H]^+^ | C_16_H_23_NO_6_ |
| 59 | 21.192 | 599.28308 | Hematoporphyrin | 32.108 | 1.7483 | [M+H]^+^ | C_34_H_38_N_4_O_6_ |
| 60 | 23.279 | 239.01845 | Cystine | 31.67 | 1.6454 | [M-H]- | C_6_H_12_N_2_O_4_S_2_ |
| 61 | 13.873 | 253.05264 | 7,4'-Dihydroxyflavone | 30.885 | 1.6527 | [M-H]- | C_15_H_10_O_4_ |
| 62 | 6.965 | 241.17105 | Pheniramine | 30.135 | 1.675 | [M+H]^+^ | C_16_H_20_N_2_ |
| 63 | 22.29 | 389.16934 | Nisoldipine | 26.405 | 1.7934 | [M+H]^+^ | C_20_H_24_N_2_O_6_ |
| 64 | 22.287 | 445.20416 | Mitoxantrone | 25.212 | 1.652 | [M+H]^+^ | C_22_H_28_N_4_O_6_ |
| 65 | 10.404 | 304.19196 | Vildagliptin | 24.968 | 1.8028 | [M+H]^+^ | C_17_H_25_N_3_O_2_ |
| 66 | 3.354 | 343.15689 | Formoterol | 24.711 | 1.8016 | [M-H]- | C_19_H_24_N_2_O_4_ |
| 67 | 3.244 | 957.49884 | Asiaticoside | 24.545 | 1.5969 | [M-H]- | C_48_H_78_O_19_ |
| 68 | 21.688 | 334.16037 | Seneciphylline | 24.25 | 1.7939 | [M+H]^+^ | C_18_H_23_NO_5_ |
| 69 | 3.687 | 353.13666 | Sudan III | 23.278 | 1.7521 | [M+H]^+^ | C_22_H_16_N_4_O |
| 70 | 22.431 | 353.21805 | Tebufenozide | 23.258 | 1.567 | [M+H]^+^ | C_22_H_28_N_2_O_2_ |
| 71 | 10.223 | 441.2757 | Norverapamil | 23.198 | 1.7495 | [M+H]^+^ | C_26_H_36_N_2_O_4_ |
| 72 | 13.407 | 321.04587 | dTMP | 22.482 | 1.5333 | [M-H]- | C_10_H_15_N_2_O_8_P |
| 73 | 9.262 | 395.03708 | Cephalothin | 21.396 | 1.7562 | [M-H]- | C_16_H_16_N_2_O_6_S_2_ |
| 74 | 3.126 | 447.2312 | hyocholic acid | 20.506 | 1.6932 | [M+H]^+^ | C_24_H_40_O_5_ |
| 75 | 22.36 | 403.22745 | Acetyl tributyl citrate | 19.632 | 1.6698 | [M+H]^+^ | C_20_H_34_O8 |
| 76 | 22.433 | 310.15598 | Sinapine | 19.229 | 1.795 | [M+H]^+^ | C_16_H_24_NO_5_ |
| 77 | 9.64 | 705.23712 | Itraconazole | 19.097 | 1.7162 | [M+H]^+^ | C_35_H_38_C_l2_N_8_O_4_ |
| 78 | 6.386 | 214.11909 | Octhilinone | 17.536 | 1.5189 | [M+H]^+^ | C_11_H_19_NOS |
| 79 | 11.913 | 349.11127 | estrone 3-sulfate | 16.334 | 1.567 | [M-H]^-^ | C_18_H_22_O_5_S |
| 80 | 3.336 | 549.27124 | Antimycin A1 | 16.057 | 1.642 | [M+H]^+^ | C_28_H_40_N_2_O_9_ |
| 81 | 18.93 | 480.0274 | T0901317 | 15.196 | 1.7281 | [M-H]^-^ | C_17_H_12_F_9_NO_3_S |
| 82 | 3.351 | 792.41742 | PROTOVERATRINE A | 14.558 | 1.5104 | [M-H]^-^ | C_41_H_63_NO_14_ |
| 83 | 22.165 | 381.20886 | Conferone | 14.488 | 1.6855 | [M+H]^+^ | C_24_H_28_O_4_ |
| 84 | 22.473 | 295.17862 | Cinchonine | 14.474 | 1.5452 | [M+H]^+^ | C_19_H_22_N_2_O |
| 85 | 22.356 | 475.22763 | Methylprednisolone succinate | 14.054 | 1.7132 | [M+H]^+^ | C_26_H_34_O_8_ |
| 86 | 3.439 | 903.44385 | Bryostatin 1 | 13.952 | 1.6343 | [M-H]^-^ | C_47_H_68_O_17_ |
| 87 | 23.613 | 147.11374 | Lysine | 13.908 | 1.7742 | [M+H]^+^ | C_6_H_14_N_2_O_2_ |
| 88 | 4.031 | 253.99969 | Ganciclovir | 13.741 | 1.5061 | [M-H]^-^ | C_9_H_13_N_5_O_4_ |
| 89 | 21.81 | 280.07037 | Nitrazepam | 12.41 | 1.5752 | [M-H]^-^ | C_15_H_11_N_3_O_3_ |
| 90 | 3.804 | 218.92876 | Dichlorvos | 12.361 | 1.7095 | [M-H]^-^ | C_4_H_7_C_l2_O_4_P |
| 91 | 22.373 | 322.14554 | Pyriproxyfen | 11.012 | 1.7591 | [M+H]^+^ | C2_0_H_19_NO_3_ |
| 92 | 7.7 | 633.27307 | Rescinnamine | 9.7823 | 1.7034 | [M-H]- | C_35_H_42_N_2_O_9_ |
| 93 | 22.502 | 429.10068 | Bispyribac | 9.6208 | 1.7646 | [M-H]- | C_19_H_18_N_4_O_8_ |
| 94 | 22.243 | 73.06526 | (2*R*,3*R*)-(-)-2,3-Butanediol | 9.3952 | 1.6792 | [M+H]^+^ | C_4_H_10_O_2_ |
| 95 | 10.522 | 253.1015 | Methyl 3,4,5-trimethoxycinnamate | 9.06 | 1.5056 | [M+H]^+^ | C_13_H_16_O_5_ |
| 96 | 3.873 | 398.27078 | Phytosphingosine-1-P | 9.0305 | 1.5312 | [M+H]^+^ | C_18_H_40_NO_6_P |
| 97 | 19.793 | 131.05399 | Quinoxaline | 8.1215 | 1.516 | [M+H]^+^ | C_8_H_6_N_2_ |
| 98 | 22.218 | 269.06354 | L-Homocystine | 7.5554 | 1.6699 | [M+H]^+^ | C_8_H_16_N_2_O_4_S_2_ |
| 99 | 22.458 | 343.22235 | 12(S)-HETE | 7.3063 | 1.7861 | [M+H]^+^ | C_20_H_32_O_3_ |
| 100 | 20.704 | 463.07941 | Myricitrin | 6.9184 | 1.5705 | [M-H]- | C_21_H_20_O_12_ |
| 101 | 11.613 | 208.07399 | N-Benzyloxycarbonylglycine | 6.8155 | 1.7926 | [M-H]- | C_10_H_11_NO_4_ |
| 102 | 11.83 | 383.23074 | Ethynodiol diacetate | 6.6311 | 1.6745 | [M-H]- | C_24_H_32_O_4_ |
| 103 | 2.915 | 633.28369 | Liensinine | 6.6102 | 1.805 | [M+H]^+^ | C_37_H_42_N_2_O_6_ |
| 104 | 22.119 | 725.21576 | ATRACTYLOSIDE | 6.3943 | 1.6569 | [M-H]^-^ | C_30_H_46_O_16_S_2_ |
| 105 | 12.209 | 316.11172 | Piplartine | 6.296 | 1.6872 | [M-H]^-^ | C_17_H_19_NO_5_ |
| 106 | 3.203 | 359.22278 | Hepoxilin A3 | 6.2006 | 1.572 | [M+H]^+^ | C_20_H_32_O_4_ |
| 107 | 8.019 | 269.04858 | Genistein | 5.9787 | 1.5992 | [M-H]- | C_15_H_10_O_5_ |
| 108 | 20.007 | 553.03357 | Ceftriaxone | 5.9256 | 1.6755 | [M-H]- | C1_8_H_18_N_8_O_7_S_3_ |
| 109 | 10.196 | 416.06848 | Bendroflumethiazide | 5.6827 | 1.5354 | [M-H]- | C_17_H_18_F_3_N_3_O_2_S_2_ |
| 110 | 21.526 | 435.21057 | Flunisolide | 5.5637 | 1.7648 | [M+H]^+^ | C_24_H_31_FO_6_ |
| 111 | 9.569 | 417.12018 | Aloin A | 4.6485 | 1.6165 | [M-H]^-^ | C21H22O9 |
| 112 | 8.274 | 356.06644 | Indomethacin | 4.2882 | 1.5766 | [M-H]^-^ | C_19_H_16_ClNO_4_ |
| 113 | 8.855 | 352.09775 | Parfumine | 4.149 | 1.5559 | [M-H]^-^ | C_20_H_19_NO_5_ |
| 114 | 8.275 | 353.10208 | sesamin | 3.6772 | 1.5765 | [M-H]^-^ | C_20_H_18_O_6_ |
| 115 | 21.032 | 330.14899 | Paroxetine | 3.5727 | 1.5387 | [M+H]^+^ | C_19_H_20_FNO_3_ |
| 116 | 8.317 | 109.0293 | Pyrocatechol | 3.5379 | 1.7622 | [M-H]- | C_6_H_6_O_2_ |
| 117 | 25.977 | 161.09216 | Daminozide | 3.5336 | 1.6371 | [M+H]^+^ | C_6_H_12_N_2_O_3_ |
| 118 | 9.117 | 312.07849 | Flunitrazepam | 3.4275 | 1.7086 | [M-H]^-^ | C_16_H_12_FN_3_O_3_ |
| 119 | 3.858 | 220.9986 | Isofraxidin | 3.424 | 1.6499 | [M-H]^-^ | C_11_H_10_O_5_ |
| 120 | 8.42 | 193.00185 | ferulic acid | 3.368 | 1.7343 | [M-H]^-^ | C_10_H_10_O_4_ |
| 121 | 11.699 | 188.02162 | Tricyclazole | 3.2408 | 1.5347 | [M-H]^-^ | C_9_H_7_N_3_S |
| 122 | 9.614 | 569.28235 | neriifolin | 3.2212 | 1.5129 | [M-H]^-^ | C_30_H_46_O_8_ |
| 123 | 8.797 | 299.05966 | Kaempferide | 3.0254 | 1.5714 | [M-H]^-^ | C_16_H_12_O_6_ |
| 124 | 22.13 | 413.12024 | Nafcillin | 2.9332 | 1.553 | [M-H]^-^ | C_21_H_22_N_2_O_5_S |
| 125 | 10.452 | 214.13986 | N-heptanoyl-homoserine lactone | 2.7971 | 1.5155 | [M+H]^+^ | C_11_H_19_NO_3_ |
| 126 | 23.895 | 401.10901 | Nobiletin | 2.7898 | 1.672 | [M-H]^-^ | C_21_H_22_O_8_ |
| 127 | 22.587 | 386.17224 | Terazosin | 2.7623 | 1.5232 | [M-H]^-^ | C_19_H_25_N_5_O_4_ |
| 128 | 9.993 | 342.9921 | Thiamine monophosphate | 2.7557 | 1.5997 | [M-H]^-^ | C_12_H_18_N_4_O_4_PS |
| 129 | 11.75 | 449.07404 | astilbin | 2.6511 | 1.6704 | [M-H]^-^ | C_21_H_22_O_11_ |
| 130 | 21.79 | 421.16159 | Morusin | 2.3856 | 1.5036 | [M+H]^+^ | C_25_H_24_O_6_ |
| 131 | 10.308 | 506.2301 | Amprenavir | 2.2959 | 1.602 | [M+H]^+^ | C_25_H_35_N_3_O_6_S |
| 132 | 19.4 | 851.25977 | Maltopentaose | 2.292 | 1.6134 | [M+H]^+^ | C_30_H_52_O_26_ |
| 133 | 17.406 | 479.15057 | Isolindleyin | 2.2813 | 1.5361 | [M+H]^+^ | C_23_H_26_O_11_ |
| 134 | 10.507 | 461.22189 | Hydrocortisone succinate | 2.2616 | 1.5101 | [M-H]^-^ | C_25_H_34_O_8_ |
| 135 | 8.587 | 356.07986 | Balsalazide | 2.2329 | 1.5661 | [M-H]^-^ | C_17_H_15_N_3_O_6_ |
| 136 | 3.272 | 175.04042 | 4-Methylumbelliferone | 2.1984 | 1.5395 | [M-H]^-^ | C_10_H_8_O_3_ |
| 137 | 14.041 | 453.15552 | Methotrexate | 2.1947 | 1.6188 | [M-H]^-^ | C_20_H_22_N_8_O_5_ |
| 138 | 19.991 | 146.06264 | Indole-3-carbinol | 2.1374 | 1.7044 | [M-H]- | C_9_H_9_NO |
| 139 | 20.734 | 262.10422 | Skimmianine | 2.0612 | 1.5709 | [M+H]^+^ | C_14_H_15_NO_4_ |
| 140 | 22.019 | 278.20837 | Venlafaxine | 0.019859 | 1.5465 | [M+H]^+^ | C_17_H_27_NO_2_ |
| 141 | 22.022 | 215.11453 | 1-Hydroxytacrine | 0.01893 | 1.79 | [M+H]^+^ | C_13_H_14_N_2_O |
| 142 | 13.461 | 1060.39282 | phytanoyl-Coenzyme A | 0.014473 | 1.7327 | [M-H]- | C_41_H_74_N_7_O_17_P_3_S |
| 143 | 14.279 | 253.04842 | Chrysophanol | 0.013143 | 1.6247 | [M-H]- | C_15_H_10_O_4_ |
| 144 | 21.793 | 173.05589 | 2-naphthoic acid | 0.010738 | 1.7738 | [M+H]^+^ | C_11_H_8_O_2_ |
| 145 | 22.038 | 345.24973 | medroxyprogesterone | 0.009864 | 1.7558 | [M+H]^+^ | C_22_H_32_O_3_ |
| 146 | 20.026 | 438.06531 | Glucocheirolin | 0.009172 | 1.6485 | [M-H]- | C_11_H_21_NO_11_S_3_ |
| 147 | 21.993 | 1341.60388 | Tubeimoside I | 0.008249 | 1.7855 | [M+H]^+^ | C_63_H_98_O_29_ |
| 148 | 24.778 | 161.97995 | 3,4-Dichloroaniline | 0.008097 | 1.7169 | [M+H]^+^ | C_6_H_5_C_l2_N |
| 149 | 22.802 | 204.13448 | Crotamiton | 0.006756 | 1.7773 | [M+H]^+^ | C_13_H_17_NO |
| 150 | 23.637 | 309.1665 | Fructoselysine | 0.006487 | 1.8121 | [M+H]^+^ | C_12_H_24_N_2_O_7_ |
| 151 | 22.055 | 252.13535 | Prosulfocarb | 0.005354 | 1.8007 | [M+H]^+^ | C_14_H_21_NOS |
| 152 | 22.766 | 409.13953 | Trifloxystrobin | 0.005339 | 1.7914 | [M+H]^+^ | C_20_H_19_F_3_N_2_O_4_ |
| 153 | 22.606 | 223.07515 | Cystathionine | 0.004328 | 1.7465 | [M+H]^+^ | C7H14N2O4S |
| 154 | 13.425 | 366.99341 | 3-O-Feruloylquinic acid | 0.004277 | 1.7119 | [M-H]- | C17H20O9 |
| 155 | 22.009 | 130.60266 | Heptanoic acid | 0.003639 | 1.7916 | [M+H]^+^ | C7H14O2 |
| 156 | 22.897 | 295.15097 | Alosetron | 0.002366 | 1.7877 | [M+H]^+^ | C17H18N4O |
| 157 | 21.623 | 207.09808 | p-methoxycinnamic acid ethyl ester | 0.001731 | 1.7857 | [M+H]^+^ | C12H14O3 |
| 158 | 22.022 | 203.15053 | ADMA | 0.00132 | 1.8078 | [M+H]^+^ | C_8_H_18_N_4_O_2_ |
| 159 | 21.515 | 332.19427 | mahanimbine | 0.000947 | 1.815 | [M+H]^+^ | C_23_H_25_NO |

**Table S8.** The differences metabolites between the PA-treated group and the untreated group.

| NO. | Average.Rt(min) | Average.Mz | Metabolite.name | FC | VIP | Adduct.type | Formula |
| --- | --- | --- | --- | --- | --- | --- | --- |
| 1 | 21.849 | 342.16809 | Naltrexone | 859.69 | 1.5411 | [M+H]^+^ | C_20_H_23_NO_4_ |
| 2 | 3.775 | 367.12094 | Curcumin | 591.25 | 1.5429 | [M-H]^-^ | C_21_H_20_O_6_ |
| 3 | 3.711 | 341.10526 | Trehalose | 532.02 | 1.5494 | [M-H]^-^ | C_12_H_22_O_11_ |
| 4 | 5.874 | 210.04355 | Dimethirimol | 177.39 | 1.5352 | [M+H]^+^ | C_11_H_19_N_3_O |
| 5 | 22.266 | 198.12511 | 2-(3-Phenylpropyl)pyridine | 141.62 | 1.5485 | [M+H]^+^ | C_14_H_15_N |
| 6 | 3.448 | 356.18707 | Glaucine | 136.33 | 1.5324 | [M+H]^+^ | C_21_H_25_NO_4_ |
| 7 | 22.368 | 558.06824 | ADP-ribose | 130.85 | 1.5437 | [M-H]^-^ | C_15_H_23_N_5_O_14_P_2_ |
| 8 | 8.312 | 453.28186 | Verapamil | 118.71 | 1.5482 | [M-H]^-^ | C_27_H_38_N_2_O_4_ |
| 9 | 10.558 | 470.2218 | Nefazodone | 86.919 | 1.5338 | [M+H]^+^ | C_25_H_32_ClN_5_O_2_ |
| 10 | 22.098 | 314.13684 | Kresoxim-methyl | 86.243 | 1.5118 | [M+H]^+^ | C_18_H_19_NO_4_ |
| 11 | 8.341 | 292.02444 | N-Acetylmuramic Acid | 84.285 | 1.5417 | [M-H]^-^ | C_11_H_19_NO_8_ |
| 12 | 22.33 | 226.15619 | PROMETON | 81.188 | 1.5193 | [M+H]^+^ | C_10_H_19_N_5_O |
| 13 | 21.677 | 665.20447 | Stachyose | 75.79 | 1.521 | [M-2H]^2-^ | C_24_H_42_O_21_ |
| 14 | 8.691 | 763.35724 | DIGITOXIN | 48.141 | 1.5236 | [M-H]^-^ | C_41_H_64_O_13_ |
| 15 | 8.273 | 845.42047 | Rifabutin | 47.884 | 1.5052 | [M-H]^-^ | C_46_H_62_N_4_O_11_ |
| 16 | 21.579 | 808.12079 | Acetyl-CoA | 47.539 | 1.5385 | [M-H]^-^ | C_23_H_38_N_7_O_17_P_3_S |
| 17 | 4.635 | 177.04044 | 2-Keto-3-deoxy-D-gluconic acid | 42.162 | 1.5337 | [M-H]^-^ | C_6_H_10_O_6_ |
| 18 | 19.712 | 300.11673 | Fenoxycarb | 41.623 | 1.5226 | [M-H]^-^ | C_17_H_19_NO_4_ |
| 19 | 15.459 | 153.01843 | 2,3-Dihydroxybenzoic acid | 40.23 | 1.519 | [M-H]^-^ | C_7_H_6_O_4_ |
| 20 | 3.787 | 322.10367 | Flutolanil | 38.732 | 1.5207 | [M-H]^-^ | C_17_H_16_F_3_NO_2_ |
| 21 | 3.126 | 447.2312 | hyocholic acid | 34.477 | 1.5041 | [M+H]^+^ | C_24_H_40_O_5_ |
| 22 | 22.267 | 175.10782 | L-Theanine | 32.335 | 1.5149 | [M+H]^+^ | C_7_H_14_N_2_O_3_ |
| 23 | 8.297 | 357.08142 | sweroside | 31.239 | 1.5127 | [M-H]^-^ | C_16_H_22_O_9_ |
| 24 | 22.462 | 216.13362 | Cycloate | 27.373 | 1.5484 | [M+H]^+^ | C_11_H_21_NOS |
| 25 | 3.677 | 313.14532 | Olanzapine | 26.641 | 1.5363 | [M+H]^+^ | C_17_H_20_N_4_S |
| 26 | 22.501 | 175.12137 | N-Methyltryptamine | 26.178 | 1.5382 | [M+H]^+^ | C_11_H_14_N_2_ |
| 27 | 10.61 | 404.15115 | Perphenazine | 24.81 | 1.5488 | [M+H]^+^ | C_21_H_26_ClN_3_OS |
| 28 | 22.356 | 475.22763 | Methylprednisolone succinate | 20.859 | 1.5498 | [M+H]^+^ | C_26_H_34_O_8_ |
| 29 | 7.7 | 633.27307 | Rescinnamine | 19.742 | 1.5125 | [M-H]^-^ | C_35_H_42_N_2_O_9_ |
| 30 | 23.015 | 253.13617 | Eremanthin | 19.535 | 1.5418 | [M+Na]^+^ | C_15_H_18_O_2_ |
| 31 | 4.653 | 245.11404 | Loxoprofen | 18.376 | 1.5042 | [M-H]^-^ | C_15_H_18_O_3_ |
| 32 | 22.324 | 214.11884 | Simetryn | 16.74 | 1.5211 | [M+NH4]^+^ | C_8_H_15_N_5_S |
| 33 | 22.29 | 389.16934 | Nisoldipine | 16.132 | 1.5074 | [M+H]^+^ | C_20_H_24_N_2_O_6_ |
| 34 | 22.243 | 73.06526 | (2*R*,3*R*)-(-)-2,3-Butanediol | 14.749 | 1.5346 | [M+H]^+^ | C_4_H_10_O_2_ |
| 35 | 3.504 | 413.12442 | Flecainide | 14.612 | 1.5526 | [M-H]^-^ | C_17_H_20_F_6_N_2_O_3_ |
| 36 | 21.019 | 276.02203 | Azathioprine | 12.559 | 1.5535 | [M-H]^-^ | C_9_H_7_N_7_O_2_S |
| 37 | 21.688 | 334.16037 | Seneciphylline | 10.623 | 1.515 | [M+H]^+^ | C_18_H_23_NO_5_ |
| 38 | 20.589 | 281.0459 | Pseudobaptigenin | 10.446 | 1.5441 | [M-H]^-^ | C_16_H_10_O_5_ |
| 39 | 8.275 | 353.10208 | sesamin | 9.8836 | 1.5021 | [M-H]^-^ | C_20_H_18_O_6_ |
| 40 | 4.303 | 187.04289 | 1-Hydroxy-2-naphthoic acid | 9.3633 | 1.5127 | [M-H]^-^ | C_11_H_8_O_3_ |
| 41 | 11.613 | 208.07399 | N-Benzyloxycarbonylglycine | 9.0216 | 1.5557 | [M-H]^-^ | C_10_H_11_NO_4_ |
| 42 | 4.303 | 186.04617 | Indoleacrylic acid | 8.955 | 1.5241 | [M-H]^-^ | C_11_H_9_NO_2_ |
| 43 | 22.858 | 613.16052 | Safflomin A | 8.9479 | 1.5398 | [M+H]^+^ | C_27_H_32_O_16_ |
| 44 | 23.095 | 466.24475 | Tobramycin | 8.8967 | 1.5114 | [M-H]^-^ | C_18_H_37_N_5_O_9_ |
| 45 | 22.502 | 429.10068 | Bispyribac | 8.3881 | 1.5003 | [M-H]^-^ | C_19_H_18_N_4_O_8_ |
| 46 | 22.218 | 269.06354 | L-Homocystine | 8.248 | 1.5418 | [M+H]^+^ | C_8_H_16_N_2_O_4_S_2_ |
| 47 | 22.269 | 305.11157 | Vicine | 8.1377 | 1.5428 | [M+H]^+^ | C_10_H_16_N_4_O_7_ |
| 48 | 15.422 | 421.15347 | Losartan | 7.616 | 1.5172 | [M-H]^-^ | C_22_H_23_ClN_6_O |
| 49 | 21.055 | 223.04269 | Sinapoyl malate | 7.4218 | 1.5104 | [M-H]^-^ | C_15_H_16_O_9_ |
| 50 | 14.056 | 310.10916 | N2,N2-Dimethylguanosine | 7.3424 | 1.5502 | [M-H]^-^ | C_12_H_17_N_5_O_5_ |
| 51 | 18.402 | 183.07504 | Triethyl phosphate | 7.2805 | 1.5537 | [M+H]^+^ | C_6_H_15_O_4_P |
| 52 | 2.777 | 412.21173 | Acetophenazine | 7.1097 | 1.5531 | [M+H]^+^ | C_23_H_29_N_3_O_2_S |
| 53 | 23.895 | 401.10901 | Nobiletin | 7.0202 | 1.5162 | [M-H]^-^ | C_21_H_22_O_8_ |
| 54 | 8.274 | 356.06644 | Indomethacin | 6.6453 | 1.5089 | [M-H]^-^ | C_19_H_16_ClNO_4_ |
| 55 | 9.117 | 312.07849 | Flunitrazepam | 6.3222 | 1.5469 | [M-H]^-^ | C_16_H_12_FN_3_O_3_ |
| 56 | 8.88 | 445.06137 | CDP-ETHANOLAMINE | 5.4258 | 1.5019 | [M-H]^-^ | C_11_H_20_N_4_O_11_P_2_ |
| 57 | 11.75 | 449.07404 | astilbin | 5.0182 | 1.5374 | [M-H]^-^ | C_21_H_22_O_11_ |
| 58 | 11.084 | 193.03629 | 1,3-Dimethyluric acid | 4.7846 | 1.548 | [M-H]^-^ | C_7_H_6_N_4_O_3_ |
| 59 | 8.797 | 299.05966 | Kaempferide | 4.4248 | 1.5205 | [M-H]^-^ | C_16_H_12_O_6_ |
| 60 | 10.311 | 392.18015 | (-)-Tylocrebrine | 4.1942 | 1.5126 | [M-H]^-^ | C_24_H_27_NO_4_ |
| 61 | 8.793 | 431.08145 | Isovitexin | 4.1535 | 1.5079 | [M-H]^-^ | C_21_H_20_O_10_ |
| 62 | 10.485 | 296.15277 | Butralin | 4.0101 | 1.5049 | [M+Na]^+^ | C_14_H_21_N_3_O_4_ |
| 63 | 8.317 | 109.0293 | Pyrocatechol | 3.9579 | 1.5092 | [M-H]^-^ | C_6_H_6_O_2_ |
| 64 | 21.167 | 223.08206 | L-CYSTATHIONINE | 3.4832 | 1.5521 | [M+H]^+^ | C_7_H_14_N_2_O_4_S |
| 65 | 18.05 | 116.07189 | N-Methyl-a-aminoisobutyric acid | 3.1681 | 1.5259 | [M-H]^-^ | C_5_H_11_NO_2_ |
| 66 | 8.783 | 313.07422 | cirsimaritin | 2.9024 | 1.5101 | [M-H]^-^ | C_17_H_14_O_6_ |
| 67 | 3.481 | 126.00188 | Isoguvacine | 2.8714 | 1.5419 | [M-H]^-^ | C_6_H_9_NO_2_ |
| 68 | 11.772 | 202.10921 | Isopentenyladenine | 2.7943 | 1.5484 | [M-H]^-^ | C_10_H1_3_N_5_ |
| 69 | 8.892 | 140.96877 | 2-Mercaptoethanesulfonic acid | 2.7191 | 1.5123 | [M-H]^-^ | C_2_H_6_O_3_S_2_ |
| 70 | 11.26 | 261.11194 | Parthenin | 2.6849 | 1.5263 | [M-H]^-^ | C_15_H_18_O_4_ |
| 71 | 15.635 | 249.10381 | Diisopropyl phthalate | 2.6828 | 1.5335 | [M-H]^-^ | C_14_H_18_O_4_ |
| 72 | 8.885 | 138.97142 | Sulfoacetic acid | 2.3843 | 1.5468 | [M-H]^-^ | C_2_H_4_O_5_S |
| 73 | 18.02 | 239.11288 | Benzyl cinnamate | 2.3578 | 1.5416 | [M+H]^+^ | C_16_H_14_O_2_ |
| 74 | 18.306 | 225.0818 | Genipin | 2.2601 | 1.5355 | [M-H]^-^ | C_11_H_14_O_5_ |
| 75 | 18.077 | 375.20236 | desoximetasone | 2.2102 | 1.5018 | [M-H]^-^ | C_22_H_29_FO_4_ |
| 76 | 12.097 | 179.05722 | PARAXANTHINE | 2.1843 | 1.5136 | [M-H]^-^ | C_7_H_8_N_4_O_2_ |
| 77 | 10.326 | 135.03081 | Threonate | 2.0346 | 1.5095 | [M-H]^-^ | C_4_H_8_O_5_ |
| 78 | 6.436 | 160.06218 | L-2-Aminoadipic acid | 2.0195 | 1.5483 | [M-H]^-^ | C_6_H_11_NO_4_ |
| 79 | 3.658 | 220.0974 | METAXALONE | 0.39361 | 1.506 | [M-H]^-^ | C_12_H_15_NO_3_ |
| 80 | 22.026 | 200.17618 | Diethylcarbamazine | 0.017361 | 1.5031 | [M+H]^+^ | C1_0_H_21_N_3_O |
| 81 | 13.656 | 575.09985 | Procyanidin B2 | 0.016434 | 1.5192 | [M-H]^-^ | C_30_H_26_O_12_ |
| 82 | 3.679 | 200.12801 | Pyrimethanil | 0.01613 | 1.5151 | [M+H]^+^ | C1_2_H_13_N_3_ |
| 83 | 21.594 | 397.24442 | Melengestrol acetate | 0.01494 | 1.5253 | [M+NH4]^+^ | C_25_H_32_O_4_ |
| 84 | 21.96 | 165.07822 | 1,5-Anhydrosorbitol | 0.011212 | 1.5132 | [M+NH4]^2+^ | C_6_H_12_O_5_ |
| 85 | 22.75 | 469.17014 | Triamcinolone acetonide | 0.0104 | 1.5074 | [M-H]^-^ | C_24_H_31_FO_6_ |
| 86 | 21.793 | 173.05589 | 2-naphthoic acid | 0.01027 | 1.5581 | [M+H]^+^ | C_11_H_8_O_2_ |
| 87 | 18.377 | 306.99286 | Cimifugin | 0.0099983 | 1.514 | [M+H]^+^ | C_16_H_18_O_6_ |
| 88 | 21.993 | 1341.60388 | Tubeimoside I | 0.0080564 | 1.5338 | [M+H]^+^ | C_63_H_98_O_29_ |
| 89 | 22.819 | 235.0784 | Didanosine | 0.0075694 | 1.5547 | [M-H]^-^ | C_10_H_12_N_4_O_3_ |
| 90 | 22.74 | 239.1153 | L-Anserine | 0.0069996 | 1.5216 | [M-H]^-^ | C_10_H_16_N_4_O_3_ |
| 91 | 22.055 | 252.13535 | Prosulfocarb | 0.0055369 | 1.5641 | [M+H]^+^ | C_14_H_21_NOS |
| 92 | 22.009 | 130.60266 | Heptanoic acid | 0.0035476 | 1.544 | [M+H]^+^ | C_7_H_14_O_2_ |
| 93 | 22.644 | 436.15707 | Fluphenazine | 0.0034857 | 1.5409 | [M-H]^-^ | C_22_H_26_F_3_N_3_OS |
| 94 | 21.634 | 338.18216 | Bitertanol | 0.003335 | 1.5388 | [M+H]^+^ | C_20_H_23_N_3_O_2_ |
| 95 | 22.732 | 325.09149 | Bilobalide | 0.0020584 | 1.5375 | [M-H]^-^ | C_15_H_18_O_8_ |
| 96 | 22.022 | 203.15053 | ADMA | 0.0015166 | 1.5457 | [M+H]^+^ | C_8_H_18_N_4_O_2_ |

**Uncropped images for key assays：**

**Control:**

**
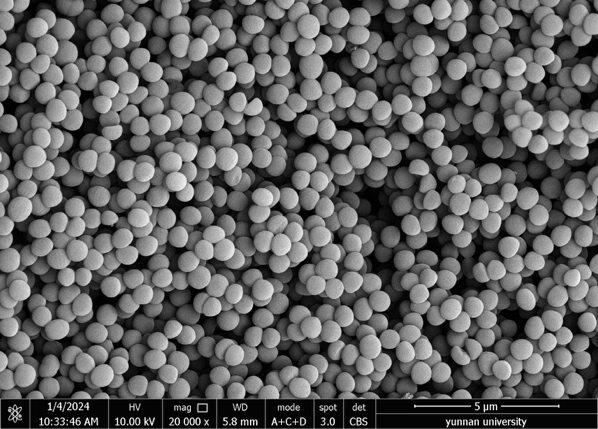

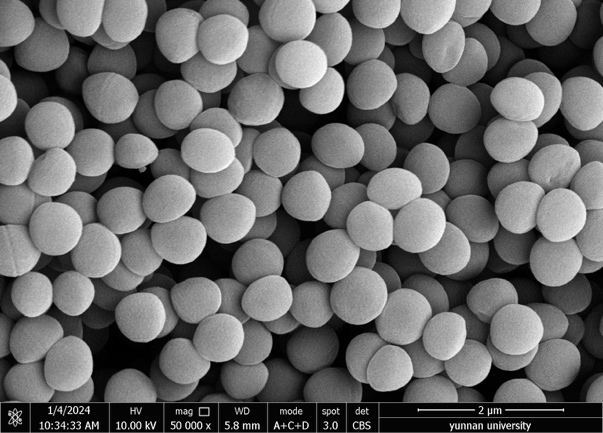
**

**MT:**

*
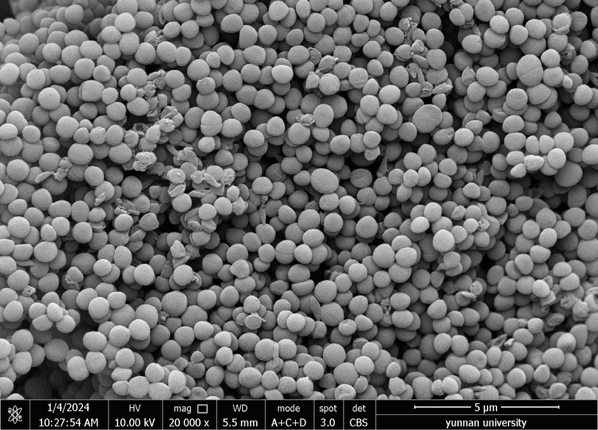
* **
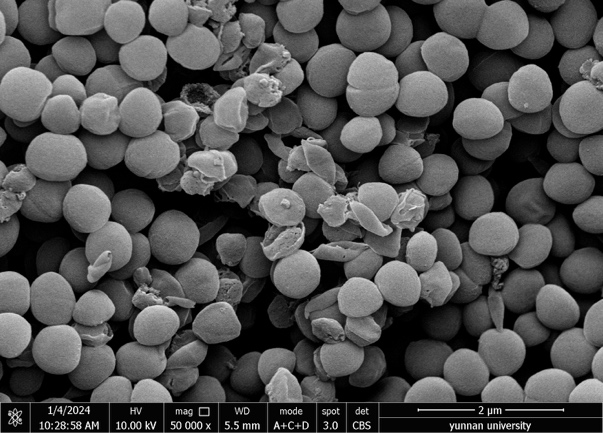
**

**PA:**

**
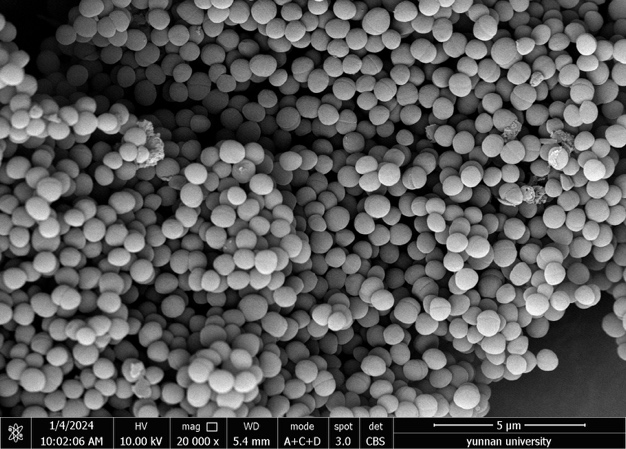

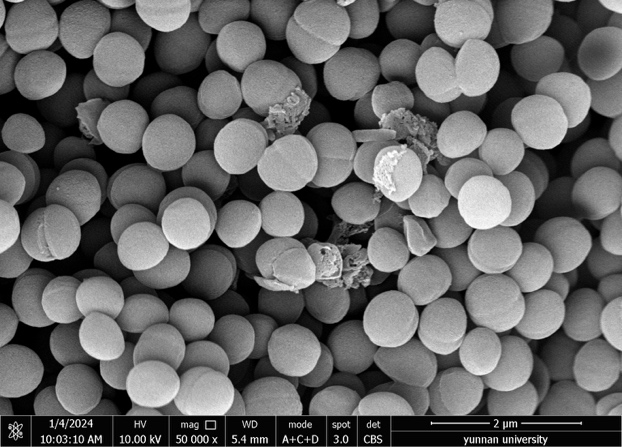
**

**Van：**

**
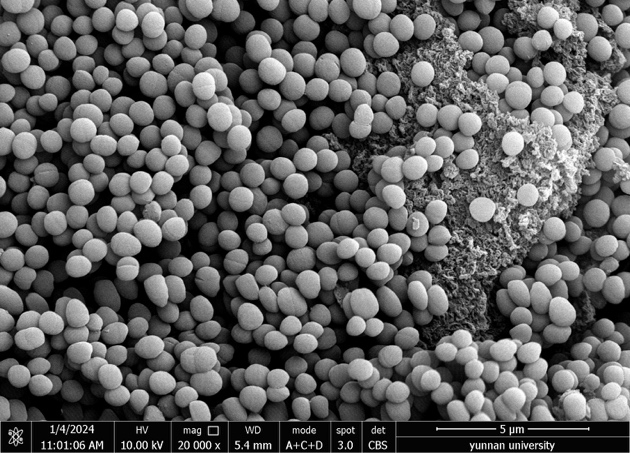

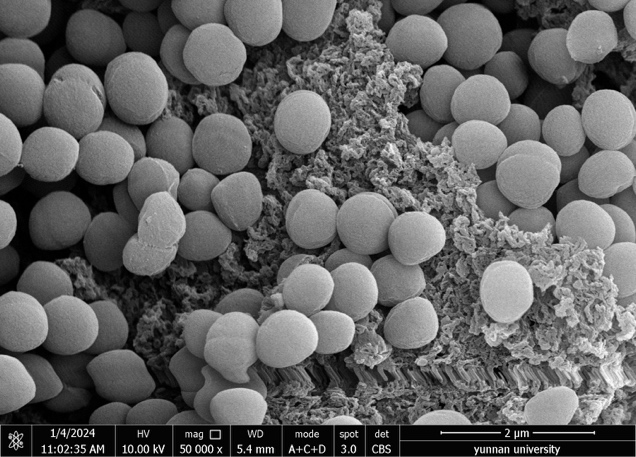
**

**References**

1. B. Wei, C. Sun, H. Wan, Q. Shou, B. Han, M. Sheng, L. Li, G. Kai, Bioactive components and molecular mechanisms of *Salvia miltiorrhiza* Bunge in promoting blood circulation to remove blood stasis, *J. Ethnopharmacol.*, 317 (2023) 116697.DOI: 10.1016/j.jep.2023.116697

2. J.R. Yu, Y.Y. Liu, Y.Y. Gao, L.H. Qian, J.L. Qiu, P.P. Wang, G.J. Zhang, Diterpenoid tanshinones inhibit gastric cancer angiogenesis through the PI3K/Akt/mTOR signaling pathway, *J. Ethnopharmacol.*, 324 (2024) 117791-117791.DOI: 10.1016/j.jep.2024.117791

3. J. Xu, K. Wei, G. Zhang, L. Lei, D. Yang, W. Wang, Q. Han, Y. Xia, Y. Bi, M. Yang, M. Li, Ethnopharmacology, phytochemistry, and pharmacology of Chinese *Salvia* species: A review, *J. Ethnopharmacol.*, 225 (2018) 18-30.DOI: 10.1016/j.jep.2018.06.029

4. X. Yu, C. Xie, L. Yu, Studies on the Chemical Constituents from *Salvia Plectranthoides* Griff, *J. Huazhong Univ. Sci. Technol.*, 31 (2002) 254-255

5. L. Xie, X.-J. Zhang, Y. Wang, Y.-F. Shi, P.-P. Wang, D. Zagal, C.-H. Li, A new anti-neuroinflammation labdane diterpenoid from *Salvia tricuspis*, *Nat. Prod. Res.*, DOI 10.1080/14786419.2023.2248541(2023).DOI: 10.1080/14786419.2023.2248541

6. G. Cao, J. Huang, Q. Zhang, K. Li, L. Shen, Chemical Constituents from *Salvia castanea*, *Chin. J. Exp. Tradit. Med. Formulae*, 23 (2017) 47-50

7. X. Li, Y. Luo, L. Wang, Y. Li, Y. Shi, Y. Cui, M. Xue, Acute and subacute toxicity of ethanol extracts from *Salvia przewalskii* Maxim in rodents, *J. Ethnopharmacol.*, 131 (2010) 110-115.DOI: 10.1016/j.jep.2010.06.012
